# Efficacy of activity tracker-based interventions and their behavioral components in promoting physical activity and reducing sedentary behavior in older adults: a systematic review of randomized controlled trials

**DOI:** 10.1186/s11556-025-00396-5

**Published:** 2026-01-12

**Authors:** Isabell Estorff, Benedict Ebert, Frieda L. Fischer, Livia Ratzlaff, Petra Wagner, Daniel Schoene

**Affiliations:** 1 Institute of Exercise and Public Health, Leipzig University, Leipzig, Germany; 2 Department of Clinical Gerontology, Robert Bosch Hospital, Stuttgart, Germany; 3 Institute of Epidemiology and Medical Biometry, University of Ulm, Ulm, Germany

**Keywords:** Aged, Older adults, Activity tracker, Physical activity, Sedentary behavior, Behavior change

## Abstract

**Background:**

Older adults often exhibit insufficient physical activity (PA) and sedentary behavior (SB), which contributes to chronic disease, functional decline, and reduced independence. From a public health perspective, low-threshold interventions are needed to promote PA and reduce SB in older adults. Wearable activity trackers offer a promising strategy by providing real-time feedback and integrating behavior change techniques (BCTs). However, evidence regarding their efficacy in older adults is still limited and inconsistent.

**Methods:**

A systematic review of randomized controlled trials (RCTs) was conducted to evaluate the efficacy of activity trackers in increasing PA and reducing SB in older adults. This review extended previous research by examining the behavioral intervention components in activity trackers. In addition, it assessed a broad range of PA and SB outcomes, categorizing them according to intervention duration to differentiate between short- (≤ 3 months), medium- (> 3–6 months), and long-term effects (> 6 months). Eligible studies met the following criteria: (a) RCT; (b) older adults (≥ 60 years or mean age ≥ 60 years minus 1 SD) not recruited for specific diseases; (c) use of an activity tracker ≥ 2 weeks; (d) comparison with at least one control group; and (e) report of PA or SB outcomes. Four databases (CENTRAL, PubMed, SPORTDiscus, Web of Science) and additional sources were screened. Risk of bias was assessed using the Cochrane RoB 2 tool.

**Results:**

Eighteen RCTs (*N* = 2,841) met the inclusion criteria. Most studies reported positive effects of activity trackers on PA, particularly regarding steps per day and intensity-related PA outcomes, with effects observed in the short- and medium-term. For SB, some studies indicated a reduction in self-reported SB, whereas no improvements were reported for objectively measured SB. Most interventions combined activity trackers with multiple BCTs, most frequently goal setting, self-monitoring, and feedback on behavior. Interventions using these components more consistently demonstrated positive outcomes, indicating that specific BCTs may facilitate behavior change.

**Conclusion:**

Activity trackers can promote PA in older adults, particularly when combined with behavioral strategies. However, their impact on objectively measured SB and long-term outcomes remains unclear. Future interventions should explicitly target SB, align BCTs with SB-related goals, and promote long-term engagement among older adults.

**Supplementary Information:**

The online version contains supplementary material available at 10.1186/s11556-025-00396-5.

## Background

Sufficient levels of physical activity (PA) are important for older adults to prevent and manage diseases and to maintain autonomy in daily living [1, 2]. To achieve health benefits, older adults are recommended to accumulate at least 150 minutes of moderate-intensity aerobic PA, or 75 minutes of vigorous-intensity aerobic PA, or an equivalent combination of both per week [3]. However, the vast majority of older adults fail to meet these recommendations, resulting in a substantial burden on individuals and society [4, 5]. In addition, a high proportion of older adults lead sedentary lifestyles, spending more than nine hours per day in sedentary activities during waking hours [6]. While physical inactivity refers to insufficient engagement in moderate-to-vigorous-intensity physical activity (MVPA), sedentary behavior (SB) is characterized by prolonged sitting or reclining during waking hours, regardless of overall PA levels. Both represent independent risk factors associated with adverse health outcomes, such as cardiovascular disease, type 2 diabetes, and frailty [3]. From a public health perspective, low-threshold interventions are necessary to increase PA and reduce SB in older adults. Walking, as the most preferred and accessible form of leisure-time PA among older adults, holds particular significance in addressing these challenges [7, 8]. In the context of this review, PA is conceptualized as habitual, self-initiated movement performed in daily life, encompassing overall activity volume and intensity (e.g., steps, walking, time spent in MVPA), rather than structured, supervised, or program-based exercise. This operational definition reflects how PA is most commonly targeted in activity tracker-based interventions in older adults.

A promising strategy to promote PA and counteract SB in older adults is the use of activity trackers. Activity trackers are wearable digital devices, such as pedometers or accelerometers, utilizing sensors to record a person’s PA. They provide continuous monitoring of individual PA levels and can offer real-time data on steps taken, distance traveled, energy expenditure, and occasionally even health metrics such as heart rate and sleep patterns [9, 10]. This feedback can support individuals in becoming more physically active and less sedentary [9]. Furthermore, activity trackers often incorporate behavior change techniques (BCTs) to motivate and sustain engagement in PA routines. These techniques include personalized goal setting, providing tailored feedback, sending motivational prompts or reminders, and employing gamification elements such as rewards or challenges [10, 11]. A key advantage of such digital tools compared to traditional face-to-face interventions is their flexible applicability, regardless of personal contact, location, or time. As a result, there is growing interest in using wearable devices to make behavior change interventions more accessible and cost-effective for older adults [12].

Meta-analyses have shown that interventions using activity trackers can increase PA in older adults, particularly steps per day, total daily PA, and MVPA [13–15], with inconsistent effects on SB [15]. However, these meta-analyses largely pooled multicomponent interventions in which activity trackers were combined with coaching, group education, or structured exercise, resulting in substantial heterogeneity and low to very low certainty of evidence. Most evidence was derived from short-term trials and heterogeneous populations, including studies focusing on specific clinical populations. SB outcomes were often underrepresented, and the specific tracker-embedded features driving behavioral change or supporting long-term maintenance remained unclear. Despite growing research on BCTs, evidence regarding which specific techniques or combinations thereof most effectively drive sustained changes in PA and SB remains weak, partly due to methodological limitations and the complex interplay of multiple BCTs within interventions [16]. Preliminary evidence suggests that self-monitoring alone may be perceived as demanding, whereas combining it with other techniques such as goal setting could be key to maintaining engagement in PA [17]. Among older adults, however, the role and relative efficacy of specific BCTs embedded in wearable activity tracker interventions remain largely unexplored.

To address these limitations, the present study conducted a narrative systematic review focusing on activity trackers as the primary intervention component in community-dwelling older adults. This approach enabled a detailed examination of behavioral features (e.g., goal setting, feedback, reminders, gamification) and their potential role in influencing PA and SB, while avoiding potentially misleading pooled estimates from highly heterogeneous data. Additionally, a broad range of PA and SB outcomes was considered to provide a more comprehensive understanding of their impact. A further objective was to assess short-, medium-, and long-term effects of activity tracker use on PA and SB in this population. By identifying contextual and design-related determinants of efficacy, this review complements existing meta-analyses and helps clarify how and under which conditions activity trackers can effectively promote everyday PA and reduce SB in older adults. Such insights are critical for informing both research and practice aimed at optimizing the implementation of activity trackers in this population [14].

## Methods

This systematic review was registered with the International Prospective Register of Systematic Reviews (PROSPERO; registration number CRD42023362309; available at: https://www.crd.york.ac.uk/prospero/display_record.php?ID=CRD42023362309). It was conducted and reported in accordance with the Preferred Reporting Items for Systematic Reviews and Meta-Analyses (PRISMA) 2020 guidelines [18]. During the conduct of this review, a meta-analysis addressing a similar research question was published [15]. In response, we adapted our protocol and refined our objective to provide a more in-depth narrative synthesis focusing on specific intervention components and contextual factors, rather than conducting a conventional meta-analysis. With regard to these factors, included studies varied substantially, rendering quantitative pooling inappropriate.

### Information source and search strategy

The following databases were searched on October 31, 2024: PubMed, Web of Science, Cochrane Central Register of Controlled Trials (CENTRAL), and SPORTDiscus. No restrictions were applied regarding publication language or time. The search strategy was developed and piloted using PubMed. After finalizing the search strategy for PubMed, it was adapted to the syntax and indexing terms of the other databases. To limit the results to randomized controlled trials (RCTs), RCT filters were applied to the searches for PubMed and Web of Science [19, 20]. In addition, reference lists of included studies and relevant review articles were screened. Trial registrations of ongoing studies were searched via CENTRAL. Moreover, Google Scholar was hand-searched for additional relevant studies using the search strings: “activity trackers” “pedometer” “older people” “older adults” “geriatric” “physical activity” “inactivity” “sedentary” “sedentariness”. The complete search strategies can be found in the supplementary material [see Additional file 1].

### Study selection, inclusion, and exclusion criteria

All identified records were imported into EndNote and de-duplicated using the method proposed by Bramer [21]. Initially, two independent reviewers screened the titles and abstracts for eligibility. Full texts of potentially eligible studies were then retrieved and assessed by two independent reviewers. Any disagreements were resolved through discussion or, if necessary, by involving a third reviewer. In cases of uncertainty, the corresponding authors were contacted at least twice via email, approximately two weeks apart. To be included in this review, studies had to be RCTs, including cluster and cross-over designs, and meet the following eligibility criteria:

#### Population

Studies were eligible if the mean age of participants was 60 years or older. If younger participants were enrolled, the mean age minus one standard deviation (SD) had to be above 60 years. Participants needed to be able to walk independently with or without a walking aid. There were no restrictions regarding the setting (e.g. community dwelling, assisted living facility, nursing home). Studies that recruited older adults due to specific medical conditions were excluded.

#### Intervention

This review included interventions in which at least one group received activity trackers as the primary intervention component. The intervention period had to last a minimum of two weeks. Studies were excluded if activity trackers represented only a minor component of a broader intervention, making it impossible to determine their relative efficacy. This applied to studies where the main intervention focused on personal coaching, such as regular interactions with an exercise coach, or educational materials, where a booklet promoting dietary and PA goal setting was the primary component, and pedometers were merely supportive tools. Similarly, interventions in which the main component consisted of group-based activities (e.g., face-to-face PA sessions or peer-led walking groups) were excluded. Group-based programs are valuable and evidence-based strategies to promote health through PA in older adults; however, as they represent a different intervention paradigm with distinct behavioral mechanisms, they were beyond the scope of this review.

#### Comparison

Studies were included if they employed at least one active or inactive control group.

#### Outcome

Studies were included if they assessed PA or SB using either objective or self-report measurements. Outcome categories were defined based on the classifications used in the primary studies. For PA, outcomes were classified into three categories: (1) Steps, such as steps per day; (2) Total PA, including total PA in minutes per day or week, walking time in minutes per day or week, and similar measures; and (3) Intensity-related PA, such as light PA (LPA, < 3 METs), moderate PA (MPA, 3–6 METs), MVPA and vigorous PA (VPA, > 6 METs) in minutes per day or week, as well as other comparable measures reported in the studies. SB outcome measures included sedentary time in minutes per day or per week, as well as any other reported indicators related to SB.

In addition, outcomes were categorized according to the duration of the intervention to differentiate between short-, medium-, and long-term effects: short-term (≤ 3 months), medium-term (> 3–6 months), and long-term (> 6 months after baseline).

### Data extraction and analysis

Descriptive study characteristics (study registration number, authors, year, country, funding, conflict of interest), sample characteristics (e.g., number, age, sex, health status, recruitment strategy of participants), intervention characteristics (e.g., components, duration, counseling frequency, BCTs, control intervention), measurement methods (e.g., accelerometer, questionnaire), time points of assessment (e.g., baseline, after completion of intervention, follow-up), and outcome data were extracted using a pre-piloted (*n* = 3) Microsoft Excel sheet. BCTs were coded according to the Behavior Change Technique Taxonomy (v1) [22]. Each component had to clearly correspond to the definitions provided in the taxonomy. A BCT was only coded if the study reported sufficient detail to justify its presence. For studies with multiple publications, information was extracted from all related documents to ensure inclusion of the most complete and accurate data. Data extraction was performed by one reviewer and independently verified by a second reviewer. Disagreements were resolved by discussion with a third reviewer. Meta-analysis was not deemed appropriate due to the large degree of heterogeneity in utilized intervention components and outcomes. Instead, the findings were synthesized narratively to allow for a detailed qualitative comparison of study outcomes, taking into account the heterogeneity in outcome and effect measures, populations, and interventions.

### Risk of bias assessment

The risk of bias was assessed using the Cochrane Risk of Bias 2 tool (RoB 2) [23]. RoB 2 evaluates five domains: (1) bias arising from the randomization process, (2) bias due to deviations from intended interventions, (3) bias due to missing outcome data, (4) bias in measurement of the outcome, and (5) bias in the selection of the reported result. Each domain was rated as ‘low risk of bias’, ‘some concerns’, or ‘high risk of bias’, which informed the overall risk of bias judgement for each outcome type. Risk of bias was assessed at the outcome level rather than the study level. Given that self-reported PA outcomes are more susceptible to recall and social desirability bias, whereas objective outcomes are generally less prone to these biases [24], outcomes were categorized into self-reported and objective measures and are reported accordingly. Further sub-categorization was not required, as outcome domains did not vary within studies or categories (e.g., objective outcomes were derived from the same device and measurement occasion, implying identical levels of missing data). Two independent reviewers assessed the risk of bias for each outcome. Discrepancies were resolved through discussion or, if necessary, by involving a third reviewer.

## Results

### Study selection

**Fig. 1:**
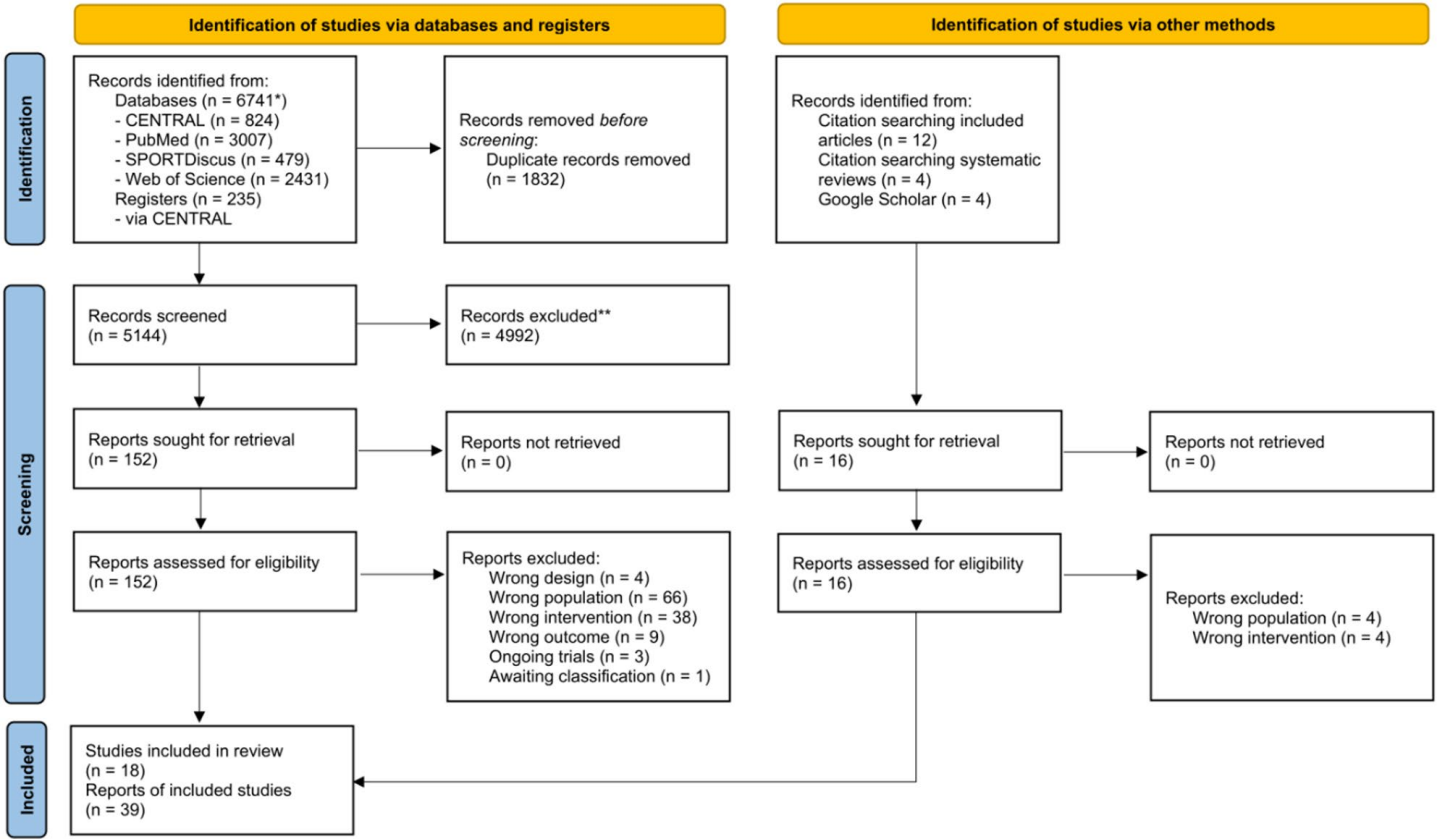
PRISMA flow diagram illustrating the search and study selection process. *excluding registry entries in CENTRAL

A total of 6,741 records were identified through database searches. An additional 20 records were identified through other sources, including reference lists of relevant reviews and meta-analyses and Google Scholar. After screening titles, abstracts, and full texts, 39 reports of 18 studies were deemed eligible and included in this systematic review (Fig. 1). The supplementary material includes the full reference list of included studies [see Additional file 3], the references of excluded studies after full-text screening categorized by reason for exclusion [see Additional files 4–7], as well as the characteristics of ongoing studies and studies awaiting classification [see Additional files 8–9].

### Study characteristics

The characteristics of the 18 included studies are summarized in Table 1. All studies were published between 2004 and 2022. A total of 2,841 older adults were included, with sample sizes ranging from 15 to 589 participants. The weighted mean age of participants was 71.25 years (range 65 to 81). On average, 74.4% (SD = 16.4%) of the participants were women.

**Table 1:** Characteristics of included studies

Author (year)	Country	Sample size	Mean age ± SD (age range) Sex	Health status	Dropout	Recruitment setting	Research design
Alley et al. (2022) [25]	Australia	Total: 243 IG1: 78 IG2: 96 CG: 76	69.3 ± 4.3 years (65–98 years) Female: 78%	Chronic disease status Yes: 89 (36.6%) No: 154 (63.4%)	*n* = 77 (28.8% at 6 weeks, 31.7% at 12 weeks, 35.4% at 24 weeks)	Not specified	IG1: web-based PA program + tailored advice based on Fitbit data IG2: web-based PA program + tailored advice based on self-reported PA *CG: no intervention
Bouchard et al. (2013) [31]	Canada	Total: 25 IG1: 9 IG2: 8 CG: 8	71.9 ± 4.5 years (not specified) Female: 52%	Chronic conditions Sum: 3.3 ± 1.6	Not specified	Not specified	*IG1: heart rate monitor to achieve PA intensity IG2: manual pulse-rate to achieve PA intensity IG3: pedometer to achieve PA intensity
Brickwood et al. (2021) [26]	Australia	Total: 117 IG1: 37 IG2: 38 CG: 42	72.4 ± 6.5 years (60.3–88.7 years) Female: 64%	Cardiovascular disease: 67 (57.3%) Metabolic disease: 56 (47.9%) Musculoskeletal conditions: 79 (67.5%) Pulmonary conditions: 22 (18.8%) Cancer: 21 (17.9%) Mental health conditions: 18 (15.4%) ≥ 2 chronic conditions: 84 (71.8%)	*n* = 18 (15.4%)	Participants of a clinical exercise program	IG1: PA intervention + activity tracker *IG2: PA intervention + telephone counselling CG: PA intervention only
Croteau et al. (2004) [32]	USA	Total: 15 IG: 7 CG: 8	80.5 ± 7.1 years (68–95 years) Female: 93%	Osteoarthritis: 14 (93.3%) Hypertension: 12 (80%) Coronary Artery Disease: 6 (40%) Cancer: 6 (40%)	Not specified	Older adults living in community-based, subsidized, assisted-living facility	IG: pedometer-based PA intervention CG: perform their normal daily activities
Croteau et al. (2007) [33]	USA	Total: 147 IG: 95 CG: 84	72.9 ± 8.8 years (55–94 years) Female: 78%	Hypertension: 71 (48.3%) Osteoarthritis: 54 (36.7%) Cancer: 26 (17.7%) Osteoporosis: 19 (12.9%) Lipid disorders: 19 (12.9%) Depression: 15 (10.2%) Coronary artery disease: 13 (8.8%) Type II diabetes: 13 (8.8%)	*n* = 31 (21.1%)	Older adults residing in a community-based or independent living facility	IG: pedometer-based PA intervention CG: wait-list
Koizumi et al. (2009) [27]	Japan	Total: 68 IG: 34 CG: 34	67.0 ± 4.0 years (60–78 years) Female: 100%	Healthy, not further specified	Not specified	Community-dwelling women	IG: PA intervention with accelerometer-based feedback on daily PA CG: continue normal daily PA patterns
Kolt et al. (2012) [34]	New Zealand	Total: 330 IG: 165 CG: 165	74.1 ± 6.1 years (not specified) Female: 54%	Taking medications for heart conditions/ hypertension: 141	*n* = 52 at end of intervention (15.76%) *n* = 60 at follow up (18.2%)	Primary care physicians	IG: PA intervention with step-related PA goals CG: PA intervention with time-related PA goals
Kwan et al. (2020) [42]	China	Total: 33 IG: 16 CG: 17	71.0 ± 9.0 years (not specified) Female: 85%	Chronic illnesses 0: 8 (24%) 1–2: 17 (52%) ≥ 3: 8 (24%)	*n* = 3 (9.1%)	Elderly community centers	IG: conventional behavior change intervention + mHealth intervention CG: conventional behavior change intervention only
Leskinen et al. (2021) [28]	Finland	Total: 231 IG: 117 CG: 114	65.2 ± 1.1 years (61.8–67.6 years) Female: 83%	Hypertension: 120 (51.9%) Diabetes: 14 (6.1%)	*n* = 5 (2.2%)	Finnish public sector employees retiring between 01/2016 and 04/2019	IG: activity tracker-based PA intervention CG: no intervention
McLellan et al. (2018) [35]	Canada	Total: 44 IG: 19 CG: 21	72.2 ± 6.3 years (not specified) Female: 70%	Not specified	*n* = 4 (9.1%)	Not specified	IG: walking program with pedometer-based feedback on walking intensity CG: walking program with no feedback on walking intensity
McMurdo et al. (2010) [36]	United Kingdom	Total: 204 IG1: 68 IG2: 68 CG: 68	77.3 ± 5.0 years (not specified) Female: 100%	Long-standing illness that limits activity Yes: 64 (30.5%) No: 146 (69.5%)	*n* = 25 (12.3%)	Local general practices	IG1: behavior change intervention only IG2: behavior change intervention + pedometer *CG: no intervention
Muellmann et al. (2019) [29]	Germany	Total: 589 IG1: 211 IG2: 119 CG: 140	69.7 ± 3.3 years (62–79 years) Female: 56.5%	Subjective health status Excellent or very good: 134 (25.4%) Good: 303 (57.3%) Less good or poor: 80 (15.1%)	*n* = 184 (31.2%)	Records of the residents’ registration offices of five communities in Northwestern Germany	IG1: web-based PA intervention IG2: web-based PA intervention + activity tracker *CG: no intervention
Roberts et al. (2019) [30]	USA	Total: 40 IG: 20 CG: 20	72.0 ± 7.4 years (not specified) Female: 60%	Moderate-to-high risk of coronary heart disease	*n* = 4 (10%)	Community-dwelling older adults	IG1: supervised exercise program IG2: supervised exercise program + activity tracker
Rowley et al. (2019) [37]	USA	Total: 170 IG1: 62 IG2: 57 CG: 51	67.3 ± 6.1 years (not specified) Female: 79.5%	Not specified	*n* = 41 (24.1%)	Community of Milwaukee, USA	IG1: pedometer-based 10.000 step intervention *IG2: pedometer-based 10.000 step intervention + website with support to increase PA systematically CG: no intervention
Slaght et al. (2017) [38]	Canada	Total: 45 IG: 23 CG: 22	70.0 years (66–77 years) Female: 71%	Successful health screening to partake in PA	*n* = 3 (6.7%)	Not specified	IG: walking program + pedometer-based feedback on walking cadence CG: walking program with no information regarding walking cadence
Sugden et al. (2008) [39]	United Kingdom	Total: 54 IG: 32 CG: 22	76.0 years (70–86 years) Female: 100%	Exclusion of persons with moderate to severe cognitive impairment (MMSE score < 18)	*n* = 9 (16.7%)	Via Scottish Primary Care Research Network	IG: theory-based PA intervention + pedometer CG: theory-based PA intervention only
Thomas et al. (2012) [40]	China	Total: 399 IG: 204 CG: 195	72.2 ± 6.0 years (not specified) Female: 66.2%	Not specified	*n* = 43 (10.8%)	Community centers for older people, which provide social and recreational services	*IG1: pedometer-based PA intervention + peer support IG2: pedometer-based PA intervention only *IG3: peer support only CG: no intervention
Yamada et al. (2012) [41]	Japan	Total: 87 IG: 43 CG: 44	75.7 ± 6.8 years (not specified) Female: 48.8%	Number of medications: 4.8	*n* = 5 (5.8%)	Not specified	IG: pedometer-based behavioral change program CG: no intervention

*CG* control group, *IG* intervention group, *SD* standard deviation

*not considered in review

Of the 18 included trials, 14 reported specific funding sources, primarily from government, university, or non-profit organizations, with no studies reporting direct industry funding. Four studies did not report specific funding information, making it unclear if they were funded. Regarding conflicts of interest (COI), 13 studies explicitly stated no COI, while one study reported COIs for two authors that were not related to the current study. Two studies did not report on either funding or COI [see Additional file 2].

### Intervention characteristics

Table 2 provides an overview of the intervention characteristics for each of the included studies. Accelerometers [25–30], pedometers [31–41], and smartphones [42] were used to track PA or SB. Accelerometers were worn on the wrists [25, 26, 28] or waist [27]. Pedometers were attached to the waist [31, 33, 36, 39], the hip [32, 38], the neck [36, 39], or the pocket of the dominant leg [41]. Smartphones were kept in a pants pocket or backpack [42]. In six studies, the wearing position of the activity tracker was not reported [29, 30, 34, 35, 37, 40]. Participants wore the activity tracker either continuously throughout the entire intervention [25–28], or during all waking hours except when participating in water-based activities (e.g., bathing, swimming) or during additional PA interventions of the study (e.g., exercise program) [30–33, 36, 39, 41]. Two studies documented non-wear time [26, 30]. Activity trackers were used to support achieving predefined PA intensities [31, 35, 38], to deliver tailored advice [25, 27–30], and to monitor daily step counts [26, 32–34, 36, 37, 39–41]. Functionally, accelerometers typically allow more detailed and continuous monitoring of PA intensity and patterns and may include more advanced features, such as gamification elements, or enhanced self-monitoring functions. In contrast, pedometers primarily record step counts. However, in the included studies, both device types were frequently combined with additional components, such as web-based programs or counseling, that introduced similar sets of BCTs beyond the device’s core functions.

**Table 2:** Intervention characteristics

Author (year)	Activity tracker	Components of the activity tracker intervention	Intervention dose
Alley et al. (2022) [25]	Accelerometer (Fitbit)	Web-based PA program with six modules of computer tailored advice based on Fitbit data delivered biweekly, action planning tool, exercise library	Duration: 12 weeks Counseling frequency: none reported
Bouchard et al. (2013) [31]	Pedometer (YAMAX Health and Sports)	Initial training session about PA guidelines and how to use the tool to help identify aerobic exercise intensity, definition of a SMART PA goal for the next eight weeks, exercise logbook to promote exercise adherence, list of locations and walking groups specific to the home surroundings of the participants	Duration: 8 weeks Counseling frequency: one after 4 weeks
Brickwood et al. (2021) [26]	Accelerometer (Jawbone UP24)	Individualized home-based exercise program, optional referral to a community-based exercise program, activity tracker, daily step goals, weekly personalized text message feedback from an accredited exercise physiologist	Duration: 12 months Counseling frequency: one per week
Croteau et al. (2004) [32]	Pedometer (Yamax Digi-Walker SW-200)	Initial counseling session (pedometer usage, goal setting, strategies to increase PA), four weeks of pedometer usage, daily step goals, weekly follow-up meetings	Duration: 4 weeks Counseling frequency: one per week
Croteau et al. (2007) [33]	Pedometer (Yamax Digi-Walker SW-200)	Initial individual meeting with a facilitator counseling, pedometer usage, daily step goals, step calendar, monthly counseling group sessions	Duration: 12 weeks Counseling frequency: one per month
Koizumi et al. (2009) [27]	Accelerometer (Kenz Lifecorder)	Accelerometer-based feedback on daily PA (steps, MVPA), feedback from researchers based on accelerometer data every two weeks	Duration: 12 weeks Counseling frequency: one every 2 weeks
Kolt et al. (2012) [34]	Pedometer (not specified)	Initial face-to-face advice on engaging in PA, PA intervention with pedometer step-related goals, telephone counseling sessions with motivational interview techniques focused on setting step-related goals led by a trained PA counselor every four weeks, logbook of daily step counts	Duration: 12 weeks Counseling frequency: one per month
Kwan et al. (2020) [42]	Smartphone applications (Samsung Health)	Conventional behavior change intervention (counseling sessions with motivational interviews, regular face-to-face and telephone support, health education, brisk walking training program), mHealth intervention (validity-tested device, personalization of goals, e-coaching, messages of praise, self-tracking, e-reminders)	Duration: 12 weeks Counseling frequency: at least once a week
Leskinen et al. (2021) [28]	Accelerometer (Polar Loop 2)	Activity tracker with multiple BCTs, daily PA goal, inactivity alerts, web-based program (Polar Flow) with feedback on the uploaded PA data	Duration: 12 months Counseling frequency: no contact during intervention
McLellan et al. (2018) [35]	Pedometer (StepsCount PiezoRx^®^)	Walking program with pedometer-based feedback on walking intensity, initial education sessions on how to use the pedometer	Duration: 6 weeks Counseling frequency: no contact during intervention
McMurdo et al. (2010) [36]	Pedometer (Omron HJ-113)	Behavior change intervention (individualized activity action plans, plans to address barriers to action, education session focusing on beliefs and motivation for walking, self-regulation PA intervention based on BCTs, monthly daily activity diaries, telephone counseling sessions with decreasing frequency) and pedometer	Duration: 6 months Counseling frequency: 11 contacts during intervention
Muellmann et al. (2019) [29]	Accelerometer (Fitbit Zip^®^)	Web-based PA intervention (website with feedback on PA goals, goal-specific rewards, opportunity to network with other intervention participants), printed brochures outlining exercises for different difficulty levels and local PA offers, weekly group meetings led by trained researchers), activity tracker to objectively track PA behavior	Duration: 10 weeks Counseling frequency: one per week
Roberts et al. (2019) [30]	Accelerometer (Fitbit Zip^®^)	Supervised center-based exercise program with aerobic and resistance exercises twice-weekly for 8 weeks, individualized cognitive-behavioral counseling that focused on reducing SB and increasing PA in daily life, activity tracker with behavioral monitoring, feedback by study team to enhance non-exercise PA	Duration: 20 weeks Counseling frequency: one per week
Rowley et al. (2019) [37]	Pedometer (Omron HJ-720ITC)	Pedometer to achieve and maintain 10,000 steps per day, interactive website with strategies to increase PA systematically (education, goal setting, self-regulation, feedback, rewards, pedometer upload, counseling guided by an ongoing discussion forum)	Duration: 12 weeks Counseling frequency: one per week
Slaght et al. (2017) [38]	Pedometer (StepRx)	On-site walking program with pedometer-based information on prescribed individualized walking cadence, visual feedback every time the participant completed one 10-minute bout of MVPA, self-report of walking time and walking intensity using a logbook	Duration: 12 weeks Counseling frequency: 10 contacts during intervention
Sugden et al. (2008) [39]	Pedometer (Omron HJ-005 or Omron HJ-113)	Theory-based PA self-regulation intervention (motivational techniques, goal setting, barrier identification), self-monitoring with pedometers and daily activity diaries, telephone counseling sessions	Duration: 12 weeks Counseling frequency: 8 contacts during intervention
Thomas et al. (2012) [40]	Pedometer (Yamax Digi-Walker SW-200)	Initial group-based face-to-face counseling with instructions and advice, pedometer with the prompt to increase the number of daily steps by 3500 extra steps a day, buddy peer support system with instructions on how to enlist support and walking partners, monthly telephone calls with supportive feedback during the first six months, monthly organized events to promote PA (e.g., organized walks)	Duration: 12 months Counseling frequency: 18 contacts during intervention
Yamada et al. (2012) [41]	Pedometer (Yamax Powerwalker EX-510)	Pedometer-based behavior change program (motivation for walking followed by goal setting, self-monitoring and feedback), instruction to increase mean daily steps by 10% each month, monthly written activity logs by participants with feedback from the research team	Duration: 6 months Counseling frequency: one per month

*BCTs* behavior change techniques, *MVPA* moderate-to-vigorous physical activity, *PA* physical activity, *SB* sedentary behavior

Activity tracker interventions were compared either against an inactive control group and/or to an active control group with a behavior change intervention (without activity tracker use), e.g., a supervised exercise program [30], a computer-based PA intervention [25, 29, 37], a home-based exercise program [26], or a peer support intervention [40]. The intervention periods ranged from four weeks up to 12 months with eight out of 18 studies (44%) evaluating an activity tracker intervention over a 12-week period [25, 27, 33, 34, 37–39, 42]. Thirteen studies used a follow-up measurement at three or six months post-intervention [25–29, 33, 34, 36–39, 41, 42].

In nearly all studies, the activity tracker was supplemented with multiple BCTs and counseling sessions during the intervention. A detailed overview of all extracted BCTs according to the BCT Taxonomy (v1) [22], including how each technique was implemented within the respective activity tracker interventions, is provided in the supplementary material [see Additional file 10]. The most frequently applied BCTs were goal setting, self-monitoring, and feedback on behavior. Goal setting was primarily used to define individualized activity goals, such as meeting PA recommendations [25, 29, 31, 38, 40], achieving daily step targets [26, 32–34, 37, 39–41], or reducing and interrupting sedentary time [25]. Three studies specified the activity goals based on pre-assessments (e.g., physical function, cardiorespiratory fitness, walking test) or previous PA levels of the participants [26, 34, 38]. In six studies, the goals were re-evaluated or increased during the intervention [31, 32, 37, 39–41]. Self-monitoring was enabled by displaying individual activity data with the activity tracker, predominantly daily steps [26, 31–34, 36, 37, 39–42], PA intensity [31], walking cadence [38], non-exercise PA [30], or multiple PA indicators (e.g., minutes, intensity, bouts) [25, 28, 29, 42]. In four studies, the activity trackers created a visual signal when participants achieved a defined activity goal [25, 28, 35, 38]. Four studies provided participants with further individualized feedback after they uploaded their activity tracker data to a web-based program [25, 28, 29, 37]. Ten out of 18 studies included additional counseling sessions with individualized feedback from the research team [27, 29, 30, 36, 39–42] or health professionals [26, 34], in addition to activity trackers. However, no differences in efficacy were observed between interventions with additional counseling and those without. 

### Measurement of PA and SB

The outcome measures used to assess PA and SB are summarized in Table 3. Sixteen studies assessed PA objectively (nine with accelerometer-based trackers, six with pedometers, one with both), while two studies relied exclusively on self-report and five combined both approaches. The most frequently used accelerometer was the ActiGraph, while Yamax pedometers were the most common pedometers. Self-reported PA was assessed with standardized questionnaires (e.g., IPAQ, PASE). Reported outcomes varied widely, with steps per day being the most common measure (10 of 18 studies), followed by total PA and activity intensities (LPA, MPA, MVPA), reported either in minutes per day, minutes per week or in 10-minute bouts.

**Table 3:** Outcome measures and results for included studies

Author (year)	Assessment method (PA & SB)	Measurement point	Outcome measures	Time effect (intervention group)	Time effect (control group)	Interaction effect (time*group)
Alley et al. (2022) [25]	Accelerometer (ActiGraph) Questionnaires (AAS, WSQ)	12 weeks	MVPA (min/week), obj. Total PA (min/week), subj. Sedentary time (min/day), obj. Sedentary time (min/day), subj.	↑ ↑* ↑* ↓*	↓ ↑* ↑ ↓	No Yes No No
Bouchard et al. (2013) [31]	Pedometer (Yamax) Questionnaire (IPAQ)	8 weeks	Total PA (MET-min/week), subj. MPA (MET-min/week), subj. Total PA (steps/day), obj. Aerobic exercise (min/week), obj. MVPA (min/week), obj.	↑* ↑* ↑* ↑* ↑	↑ ↑ ↑ ↑ ↑	Yes No No No No
Brickwood et al. (2021) [26]	Accelerometer (ActivPal) Questionnaire (AAS)	12 months	Total PA (steps/day), obj. PA (min/week), subj. PA (MET-min/week), subj. Sedentary time (min/day), obj.	↑ ↓ ↓ Not reported	↓* --- ↑ Not reported	No No No Not reported
Croteau et al. (2004) [32]	Pedometer (Yamax) Questionnaire (PASE)	6 weeks	Total PA (steps/day), obj. Total PA (score/week), subj.	↓ ↓	↑ ↑	No No
Croteau et al. (2007) [33]	Pedometer (Yamax)	12 weeks	Total PA (steps/day), obj.	↑*	↓	Yes
Koizumi et al. (2009) [27]	Accelerometer (Kenz Liefcorder)	12 weeks	Total PA (steps/day), obj. MPA (min/day), obj.	↑* ↑*	↓ ↓	Yes Yes
Kolt et al. (2012) [34]	Questionnaire (AHSPAQ)	12 weeks	Total PA in leisure time (min/week), subj. MPA in leisure time (min/week), subj. Walking in leisure time (min/week), subj. Total PA by walking (min/week), subj.	↑* ↑* ↑* ↑*	↑ ↑ ↑ ↑	No No Yes No
Kwan et al. (2020) [42]	Accelerometer (ActiGraph) Questionnaire (PASE)	13 weeks	PASE (score/week), subj. Walking time (min/day), obj. Walking (steps/day), obj. Walking fast (min/day), obj. MVPA (min/week), obj.	↑* ↑* ↑* ↑* ↑*	↑ ↓ ↓ --- ↑	Not reported Not reported Not reported Not reported Not reported
Leskinen et al. (2021) [28]	Accelerometer (ActiGraph)	12 months	Total PA (min/day), obj. LPA (min/day), obj. MVPA (min/day), obj.	↓ ↓ ↓	↓ ↓ ↓	No No No
McLellan et al. (2018) [35]	Pedometer (PiezoRx^®^ Step Count)	6 weeks	MVPA in 10 min bouts (min/week), obj.	↑*	↓*	Yes
McMurdo et al. (2010) [36]	Accelerometer (RT3)	6 months	Accelerometer counts (counts/day), obj. Total PA (min/day), obj.	↓ ↑	↑ ↓	No Not reported
Muellmann et al. (2019) [29]	Accelerometer (ActiGraph)	12 weeks	MVPA (min/day), obj. MVPA in 10 min bouts (min/week), obj. Sedentary time (min/day), obj. Sedentary time in 30 min bouts (min/week), obj.	↑ ↑ ↓ ↓	↓ ↓ ↓ ↓	No Yes No No
Roberts et al. (2019) [30]	Accelerometer (ActiGraph)	20 weeks	Total PA (steps/day), obj. Total sedentary time (min/day), obj.	↑ ↑	↓ ↑	No No
Rowley et al. (2019) [37]	Pedometer (Omron)	12 weeks	Total PA (steps/day), obj.	↑*	---	Yes
Slaght et al. (2017) [38]	Accelerometer (Philips Respironics) Pedometer (StepRX Ontario)	12 weeks	MVPA in 10 min bouts (min/week), obj. Total MVPA (min/week), obj. Total PA (steps/day), obj.	↑* ↑* ↑	↓* ↓* ↓*	Yes Yes Yes
Sugden et al. (2008) [39]	Accelerometer (RT3)	12 weeks	Total walking (counts/day), obj.	↓	↓	No
Thomas et al. (2012) [40]	Questionnaire (IPAQ)	12 months	Total PA (MET-min/week), subj.	↑*	Not reported	No
Yamada et al. (2012) [41]	Pedometer (Yamax)	6 months	Total PA (steps/day), obj.	↑	↑	Yes

*AAS* Active Australia Survey, *AHSPAQ* Auckland Heart Study Physical Activity Questionnaire, *IPAQ* International Physical Activity Questionnaire, *MET* metabolic equivalent of task, *min* minutes, *obj.* objective, *PA* physical activity, *PASE* Physical Activity Scale for the Elderly, *SB* sedentary behavior, *subj.* subjective, *WSQ* Workforce Sitting Questionnaire

↑ = increase, ↓ = decrease, --- = no effect, * = significant effect

Four studies measured objective SB outcomes using ActiGraph [25, 29, 30] and ActivPal [26], in addition to PA outcomes. Alley et al. (2022) also reported subjective SB outcomes assessed with the Workforce Sitting Questionnaire (WSQ) [25]. All four studies assessed sedentary time in minutes per day. In addition, SB was reported as sedentary time per week calculated in bouts of at least 30 minutes [29]. 

### Risk of bias assessment

**Fig. 2:**
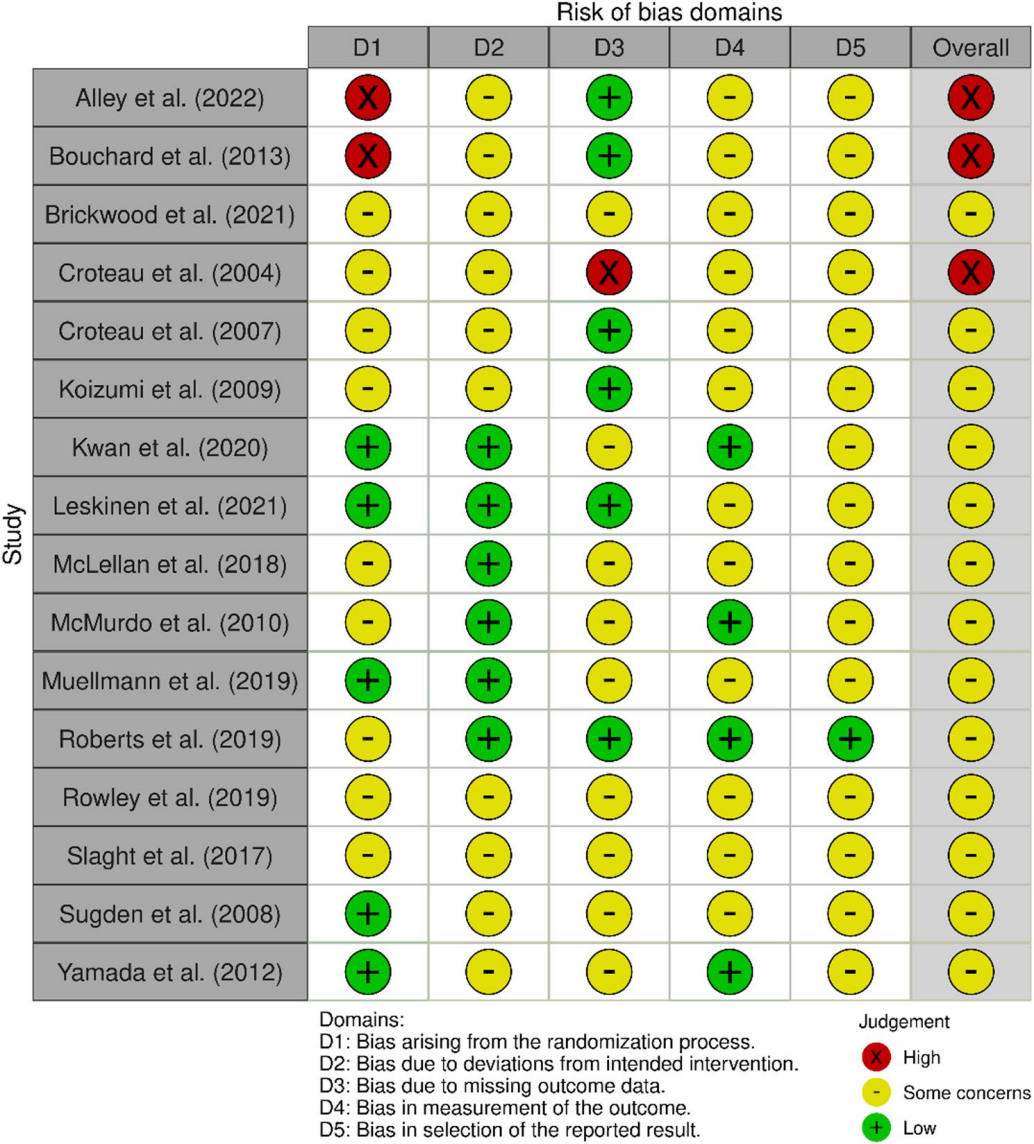
Risk of bias summary for studies using objective outcome measures

**Fig. 3:**
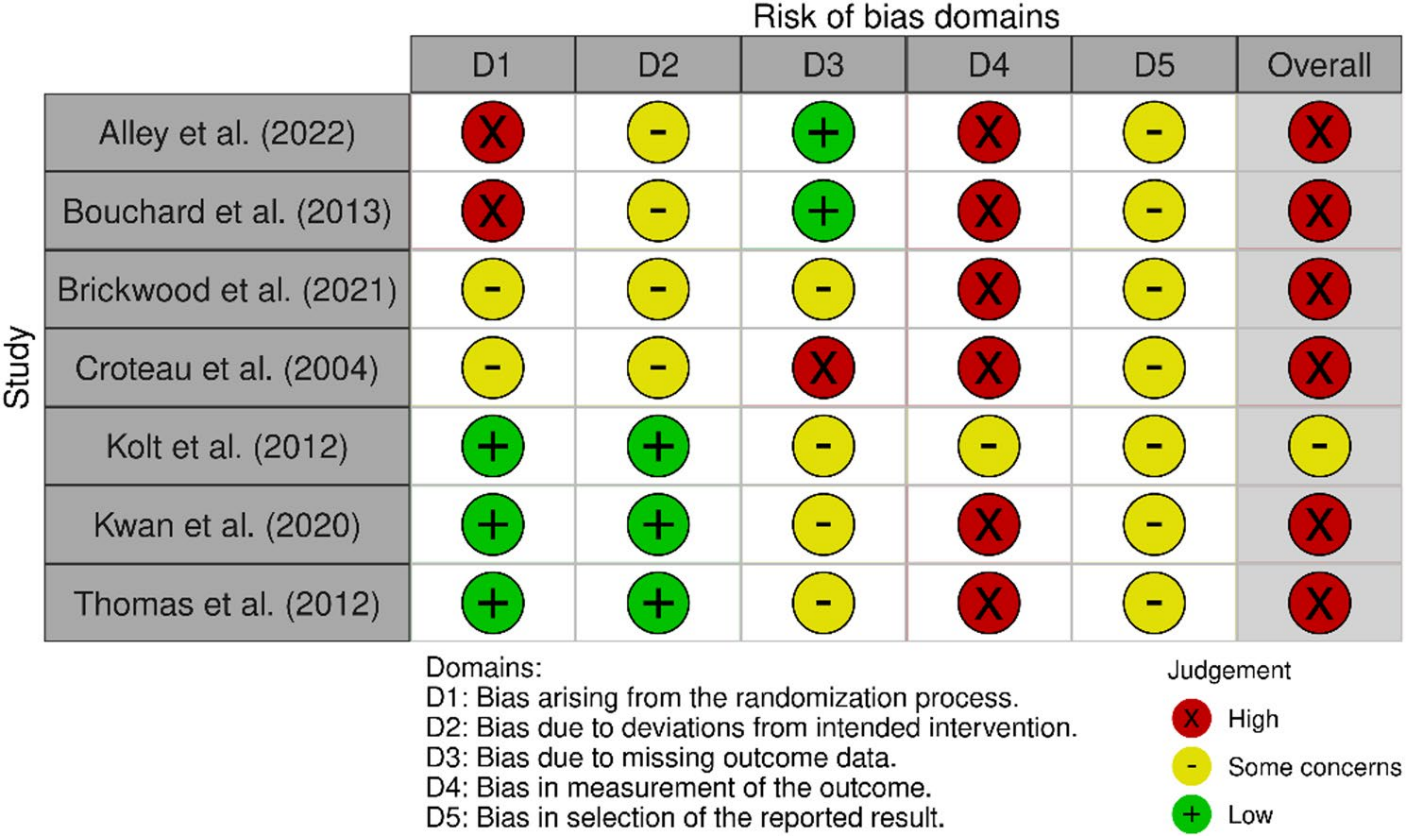
Risk of bias summary for studies using self-reported outcome measures

**Fig. 4:**
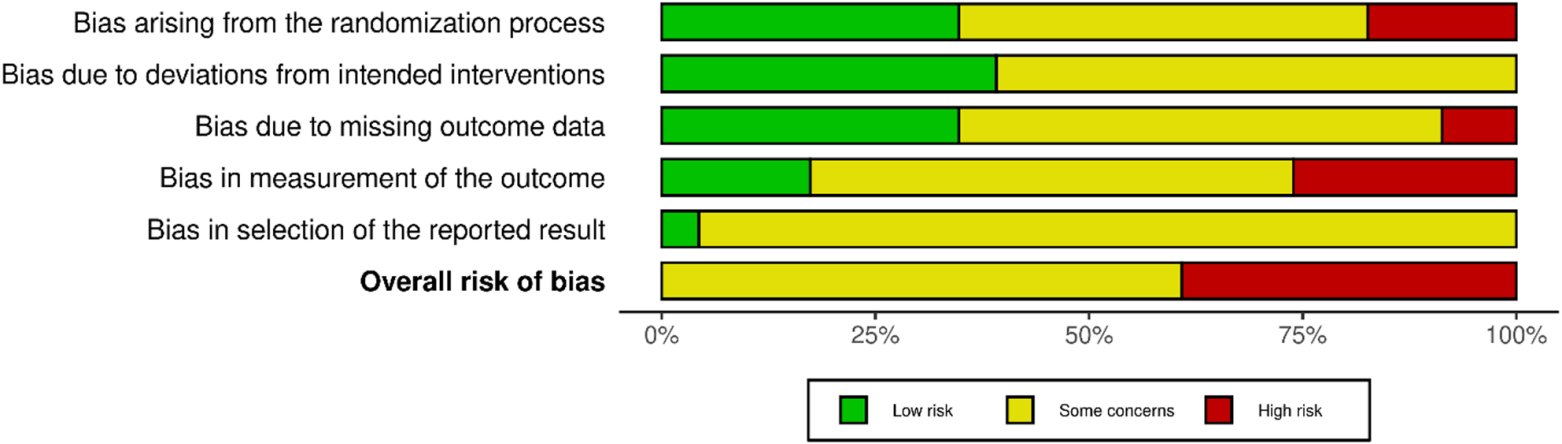
Risk of bias graph (all studies)

The risk of bias assessments are shown in Figs. 2, 3, and 4. Three of the sixteen overall ratings from the objective PA outcome category and six of seven from the self-reported PA outcome category were classified as having a high risk of bias. Reasons for a high risk of bias rating included inadequate randomization [25, 31], missing outcome data [32, 33], and knowledge of the assigned intervention may have influenced participants’ self-reported outcomes [24, 38, 40]. With the exception of the study by Roberts et al. (2019) [30], all other studies were judged to have some concerns due to bias in the selection of reported results, as no protocol or sufficiently comprehensive trial registry entry could be identified. No study was judged to be at a low risk of bias.

### PA outcomes and behavioral components of activity tracker interventions

The detailed outcome measures and statistical results for all included studies (time effect intervention group, time effect control group, interaction effect) are presented in Table 3.

#### Steps

Seven of 10 studies reported significant increases in daily steps of activity tracker interventions over time and/or in comparison to control groups [27, 31, 33, 37, 38, 41, 42]. The increase in daily steps ranged from 639 [32] to 3,016 [37] additional steps on average, and up to 3,778.9 steps at the median [42]. Positive effects on steps per day were primarily observed in the short- and medium-term, while the only long-term trial did not find improvements [26]. Positive effects were most often seen when the activity tracker use was combined with BCTs such as goal setting [27, 31, 33, 37, 38, 41, 42], self-monitoring [31, 33, 38, 41, 42], and feedback on behavior [27, 33, 37, 38, 41, 42], particularly focusing on step-based goals [33, 37, 41]. However, studies that did not find changes in the number of steps between groups also utilized BCTs [26, 30, 32].

#### Total PA

Ten studies reported outcomes of activity tracker interventions on total PA, all of which incorporated goal setting and self-monitoring as BCTs. Assessment methods varied, including minutes per day [28, 36], minutes per week [25, 26], MET-minutes per week [26, 31, 40], composite scores [32], walking time or active leisure time [34, 39, 42]. Five studies reported significant increases in self-reported PA [25, 26, 31, 34, 40]. For example, Alley et al. [25] reported a 34% greater increase in self-reported PA in the activity tracker group than in controls at 6 weeks. Bouchard et al. [31] observed a median increase from 198 to 318 minutes per week (*p* = 0.003). Two studies found significant increases in MET-minutes per week [31, 40], with Thomas et al. [40] showing 1260–1820 more MET-minutes per week at 12 months compared with decreases in controls. Walking time also increased in two studies [34, 42]. In contrast, Brickwood et al. [26] reported declines in self-reported minutes per week over 12 months, although the tracker group still showed the highest activity levels compared to telephone counseling or usual care. Three studies implemented long-term interventions (≥ 6 months) on total PA [26, 28, 40], but only Thomas et al. (2012) [40] reported significant improvements.

#### Intensity-related PA

Eight studies reported intensity-related PA outcomes, mostly on MVPA measured objectively in minutes per day [28, 29], minutes per week [25, 31, 38, 42], or in 10-minute bouts per week [29, 35, 38]. Three studies reported outcomes related to LPA or MPA [27, 28, 31]. Results were mixed. Muellmann et al. [29] found a small but significant increase in MVPA measured in minutes per day after a 12-week intervention period, whereas Leskinen et al. [28] reported no change after 6 months. All four studies that evaluated MVPA in minutes per week showed an increase over time (eight to 13 weeks), although this was only significant in two studies [38, 42]. Studies assessing MVPA in 10-minute bouts also favored activity tracker groups, showing increases compared to decreases in controls [29, 35, 38]. LPA and MPA increased significantly over time [27, 31]. Activity trackers were used to support achieving predefined PA intensities, and showed positive effects for increasing MVPA [35, 38] and LPA [27, 31]. Notably, positive effects on MVPA were supported by digital goal-specific rewards via apps or online platforms [29, 42]. All reported effects on intensity-related PA outcomes were based on short- or medium-term interventions; none of the studies examined the long-term effects of activity tracker use on intensity-related PA.

### SB outcomes and behavioral components of activity tracker interventions

Four studies examined SB, with mixed results [25, 26, 29, 30]. Brickwood et al. (2021) did not report results on SB, although their protocol stated SB in minutes per day as an outcome [26]. Roberts et al. (2019) observed a modest, though non-significant, reduction of about 48 minutes per week in the activity tracker + exercise group (walking, resistance training, balance and stretching) compared with exercise alone [30]. Alley et al. (2022) found no effect of activity trackers on objectively measured SB, while self-reported sitting time decreased in the activity tracker group but did not remain significant in sensitivity analyses [25]. Similarly, Muellmann et al. (2019) found no significant change in total sedentary time per day. However, when considering total weekly time accumulated in prolonged sedentary bouts of at least 30 minutes, the activity tracker group showed a small decrease in weekly sedentary time of -16 minutes (95% CI: −179 to 146), whereas the web-based PA intervention group exhibited an increase. Although the differences favored the activity tracker group, the results were not statistically significant [29]. Overall, while some studies indicated a potential reduction in sedentary time, particularly based on self-reported measures, the results were inconsistent, and the effects rarely reached statistical significance. Reported interventions [25, 29, 30] applied several BCTs, including goal setting, action planning, self-monitoring, and feedback on behavior. None of these interventions used inactivity alerts to interrupt prolonged sitting. SB was evaluated only in the short-term (12 weeks).

## Discussion

### Summary of evidence

This systematic review analyzed 18 RCTs with a total of 2,841 participants, examining the efficacy of activity trackers in promoting PA and reducing SB in older adults. A broad range of PA and SB outcomes was considered to provide a comprehensive understanding of their impact, with a particular focus on the behavioral components integrated into the interventions. Furthermore, the short-, medium-, and long-term effects of the interventions were examined. Overall, activity trackers showed effects in increasing PA in the short- and medium-term, particularly steps per day and intensity-related PA outcomes. For reducing SB in older adults, the included studies indicated a possible short-term reduction in self-reported SB, whereas small and insignificant changes were observed for objectively measured SB.

### Efficacy of activity tracker interventions in promoting PA and behavioral mechanisms

Activity tracker interventions were generally effective in increasing daily steps, with several studies reporting improvements ranging from several hundred to over 3,000 steps per day. Previous longitudinal studies have shown that improving daily steps by 1,000 has a positive effect on health and can reduce mortality risk [43]. Nevertheless, a few studies observed no significant changes, indicating variability in intervention efficacy that could stem from differences in intervention duration, population characteristics, or motivational components. Regarding the BCTs used, no clear differences were observed compared to the studies with significant increases in steps. One of the unsuccessful studies had a very short intervention period of only four weeks [32], which may be too short to achieve an increase in PA. In another study, the group receiving activity trackers did not increase their daily steps, but maintained them, while the group receiving usual care showed a decline after 12 months [26]. While this does not reflect a significant increase in PA, maintaining activity levels over time can still be considered a positive outcome. Given the health benefits of sufficient PA, a decline in PA may contribute to reduced physical capacity, subsequent lower mobility, and a decreased ability to manage daily tasks independently [44].

The effects on total PA were mixed across studies and measurement approaches. While some studies reported significant increases, particularly in MET-minutes per week, others found only nonsignificant trends or even declines over time. One study noted a decline in self-reported PA over a 12-month period [26], highlighting the limitations of self-report measures and potential PA decline over extended periods when feedback and motivation might diminish. The mixed results indicate that while activity trackers can foster short-term increases in PA, sustained engagement over time remains a challenge, possibly requiring additional intervention components. Furthermore, significant improvements were noted for leisure-time PA and walking time in certain studies [34, 42], supporting the idea that activity trackers may particularly enhance walking behavior and recreational PA in older adults. These forms of PA are often more accessible and achievable for older adults [45] and may offer a viable pathway for increasing overall activity levels in this population. However, another study measuring walking in counts per day did not reveal any statistically significant positive effects of the activity tracker on PA [39]. This may be due to the relatively small sample size, which limited the study’s ability to detect any meaningful associations, and the 100% female sample. Given the exclusive focus on women in the study, gender-specific differences in preferences and motives among older adults using activity trackers may have influenced the intervention outcomes [46].

For MVPA, the evidence suggests that activity trackers may help older adults engage in recommended intensity levels, particularly when activity is structured in short, time-defined bouts (e.g., 10 min), which may be more feasible for older adults to integrate into daily routines. These findings underscore the potential of activity trackers to foster adherence to recommended PA intensities. However, increases in MVPA were often modest and sometimes nonsignificant, reflecting the challenges of engaging older adults in more vigorous forms of PA. Supporting this, a meta-analysis demonstrated that wearable activity trackers are effective in increasing weekly MVPA and total daily PA, although their efficacy is more pronounced in participants younger than 70 years compared to those aged 70 or older [15]. Liu et al. (2020) also highlighted that interventions were less effective in participants with a mean age of 80 years or older, which aligns with the observation of age-related challenges in achieving PA increases [13].

The application of BCTs varied considerably across the included studies, which may explain some of the heterogeneity in PA outcomes. Overall, the most frequently employed BCTs were goal setting, self-monitoring, and feedback on behavior. These strategies likely enhanced participants’ awareness and motivation by providing clear targets and ongoing reinforcement, thereby facilitating self-regulation. This aligns with previous findings that emphasize the efficacy of step-count feedback and goal setting [13, 47] and is supported by self-regulation and control theory frameworks, which describe feedback loops between goal setting, behavior monitoring, and behavioral adjustment [48]. For example, Michie and colleagues found that self-monitoring combined with another control theory-based BCT was most effective in improving health-related behavior [49]. Some interventions also incorporated educational components such as information about health consequences [28, 29, 37, 39] and instruction on how to perform a behavior [29, 31, 35, 38, 40]. These strategies aimed to enhance participants’ knowledge about the benefits of PA and provide practical guidance on how to achieve recommended activity levels (e.g., advice about the health benefits of increasing PA verbally or in pamphlet or instruction on walking cadence). Interestingly, all interventions that included instruction on how to perform a behavior and, in some cases, practice-based elements demonstrated significant improvements in PA outcomes [29, 31, 35, 38, 40]. While these techniques were typically implemented alongside other BCTs such as goal setting, feedback, and self-monitoring, making it difficult to isolate their specific effects, they may have played an important supportive role by strengthening self-efficacy and translating behavioral intentions into action among older adults. Less frequently used BCTs included rewards, prompts and cues, and social support. Among the interventions that significantly increased MVPA, two studies incorporated digital rewards via apps or online platforms, suggesting that such extrinsic incentives may enhance engagement, particularly in the short term [29, 42]. These effects may be explained by both behavioral and motivational mechanisms: extrinsic rewards can activate the brain’s reward system and increase the perceived value of the behavior in the short term [50], especially in the early stages of behavior change. From a theoretical perspective, such external incentives may support the initiation of behavior by enhancing perceived competence and reinforcing autonomy-supportive environments, as described in Self-Determination Theory [51].

However, complex interventions that include multiple BCTs and digital components may also pose challenges. For example, the study by Muellmann et al. (2019) reported a dropout rate exceeding 30%, in an intervention involving multiple BCTs and regular weekly participant contact over 10 weeks, suggesting that highly multifaceted interventions may overwhelm some older adults [29]. In general, a higher proportion of participants dropped out when a website or computer-based platform was used as part of the intervention [25, 29, 37], indicating that digital literacy and usability concerns remain a barrier in this age group. Thus, while combining multiple BCTs can strengthen intervention efficacy, future programs should carefully balance complexity with accessibility. Providing structured guidance, low-threshold technical support, and simplified user interfaces may help mitigate dropout and ensure sustained engagement. Furthermore, strategies such as social support and action planning, which are associated with long-term behavior change [52], were rarely embedded as features within the activity trackers themselves. Incorporating such elements directly into the device or its accompanying app may help sustain engagement and promote lasting behavior change.

The duration of the interventions varied across studies, ranging from four weeks to 12 months, with 12 weeks being the most common intervention length. The results demonstrate that significant increases in PA can be achieved using activity trackers during this time. In contrast, shorter and longer intervention periods of four weeks or 12 months were less effective in improving PA behaviors [26, 28, 32]. A duration of four weeks may simply be too short to initiate and consolidate meaningful behavior change, whereas longer durations may exceed the motivational capacity of low-threshold interventions such as activity trackers alone. Without additional support components, engagement and adherence tend to decline over time, which may explain the lack of sustained effects in long-term interventions.

### Efficacy of activity tracker interventions in reducing SB and behavioral mechanisms

The efficacy of activity trackers in reducing SB among older adults remains inconclusive due to the small number of included studies and the mixed findings across them. While some evidence based on self-reported measures suggests a potential reduction in SB, objective assessments generally did not confirm these effects [25, 29, 30]. This aligns with a previous systematic review that found no effect of activity trackers on SB, although that review did not focus specifically on older adults [53]. In contrast, Wu et al. (2023) reported a small but significant reduction in sedentary time in their meta-analysis of wearable activity tracker interventions targeting both PA and SB in older adults [15]. Similarly, a review focusing exclusively on interventions to reduce SB in community-dwelling older adults, regardless of intervention type, found only small and inconsistent effects and overall low certainty of evidence [54].

Consistent with this evidence, several studies in our review found small reductions in SB favoring activity tracker interventions, though without statistically significant effects in objective data. Activity tracker interventions tended to show greater improvements in SB compared to those without [25, 29, 30]. This suggests that the use of activity trackers may have small effects by increasing older adults’ awareness of their sitting habits and encouraging behavioral adjustments [25]. Even modest reductions in SB could be meaningful in this context, considering that older adults spend 60 to 80% of their waking hours sedentary [30] and research has shown that any reduction in sitting time may be beneficial for certain health outcomes, such as type 2 diabetes and mortality risk [55]. However, the relevance of some of the modest changes (less than 10 min per day) needs to be questioned and requires further research.

A notable issue concerns the discrepancy between self-reported and objectively measured SB. While some studies reported improvements based on self-report following activity tracker use [25], objective measurements consistently failed to demonstrate significant reductions [25, 29, 30]. These inconsistencies point to the potential influence of social desirability bias in self-reported SB [25], underlining the importance of including objective measures when evaluating intervention outcomes. For instance, Alley et al. (2019) even observed an increase in objectively measured SB in both intervention and control groups, suggesting that the change was unrelated to the intervention.

There are several possible reasons why activity trackers were ineffective at reducing SB. First, none of the included studies focused specifically on SB, as their primary focus was PA [25, 29]. This is supported by the findings of a systematic review and meta-analysis that found no intervention effects on SB outcomes for either interventions targeting PA or interventions targeting both PA and SB in adults [56]. Although strategies to reduce SB were implemented, the intervention components targeting SB were probably insufficient [25]. To improve efficacy, BCTs incorporated into activity trackers should be explicitly tailored to SB instead of being transferred from PA interventions. As highlighted in a recent scoping review, the type of goal setting is crucial: setting specific goals to increase sit-to-stand transitions was found to be more effective in increasing the number of breaks in SB than general goals to reduce total sitting time [57]. Accordingly, other BCTs, such as feedback on behavior, should be adapted to focus specifically on SB patterns (e.g., duration of uninterrupted sitting, number of daily standing breaks) rather than general PA indicators. This specificity may help to better target habitual sedentary routines and promote meaningful behavior change in older adults. To increase efficacy, future interventions may also need to include components such as social support and overall more frequent feedback on SB [25, 29]. Secondly, the methodological limitations of the studies may have influenced the efficacy of the interventions. These limitations included underpowered study designs [29, 30], the recruitment of already physically active older adults [29], high dropout rates [29], and the intervention duration [25, 29].

The intervention duration required to achieve a change in SB was reported differently. While Alley et al. (2022) described the 12-week intervention period as potentially too long [25], Muellmann et al. (2019) described the 10-week intervention period as potentially too short [29]. However, meta-analyses in this context suggest that activity trackers (average reduction of 7.54 min SB per day) [15] or digital interventions in general (average reduction of 41.28 min SB per day) [58] can improve SB mainly in the short term with an intervention period of up to 12 weeks.

### Strengths and limitations

A key strength of this review lies in its comprehensive approach to analyzing a wide range of PA and SB outcomes. In contrast to previous reviews, which often focused narrowly on metrics such as steps per day [14], we examined multiple PA measures, including total PA in minutes per day and week, active leisure time, intensity-related PA outcomes such as LPA, MPA, and MVPA, as well as SB outcomes. Notably, our review is one of the few to incorporate SB as a primary outcome, addressing a gap in literature where the relationship between activity trackers and SB has been underexplored. This broader scope provides a more holistic understanding of the impact of activity trackers on behavior change in older adults. However, this broad coverage also introduced substantial heterogeneity across outcomes and measures, which precluded a formal meta-analysis.

This review also has limitations that should be considered when interpreting the results. Although our search strategy was designed to capture interventions targeting habitual PA in older adults, it primarily identified studies focusing on self-initiated PA targeted by activity tracker-based interventions, which are typically reflected by ambulatory activity and/or activity intensity (e.g., steps, walking, MVPA). The MeSH term ‘Exercise’ and specific activity modalities (e.g., cycling, swimming, gardening) were not included in the search strategy. Therefore, studies emphasizing structured exercise or non-ambulatory forms of habitual PA may have been missed, although multiple databases and citation tracking were used to mitigate this risk. Furthermore, the literature search was not repeated after refinement of the research focus toward behavioral components, as the core inclusion criteria remained unchanged. Since the refinement did not alter the core inclusion criteria, we consider it unlikely that relevant studies were missed. The review focused on peer-reviewed literature to ensure methodological quality. However, emerging open science publications and preprints were not included, which may have led to omission of some recent studies. Sample sizes were often small, increasing the risk of small-study bias and potentially limiting the generalizability of the findings. The type of activity tracker used also influences the effects of the studies, depending on the level of technology, ranging from basic technologies to smart devices. While objective measurement with activity trackers carries the risk of not fully capturing PA, as non-ambulatory movements such as cycling or swimming are often not recorded [59], the exclusively subjective recording of PA in two studies may lead to bias due to overestimation [24], particularly in populations where recall accuracy may vary with age-related subtle cognitive changes. Another potential limitation is that the range of implemented BCTs may be underestimated, which may reflect either limited use or incomplete reporting in the studies. Moreover, the overall risk of bias could not be assessed as ‘low’ for any of the studies analyzed, which reduces confidence in the validity and reliability of the findings. This limitation underscores the need for future research with robust study designs that are sufficiently powered and of higher methodological quality. Such research would provide clearer evidence on the efficacy of activity trackers in the population of older adults.

### Implications for practice, policy, and future public health research

In practice, activity tracker interventions should balance simplicity, to facilitate initial adoption and usability, with sufficient support, such as guidance and feedback, to promote sustained engagement in older adults. Policies should focus on integrating activity tracker-based interventions into community and healthcare settings, making them accessible and affordable for this population. Future public health research should explore strategies to reduce SB, investigate the long-term efficacy of activity trackers, and identify the optimal combination of BCTs to support sustainable behavior change. While tailoring interventions to diverse older adult populations, considering factors such as age, gender, health status, and digital literacy, is important for feasibility and adherence, such individualization should be balanced with methodological rigor to ensure robust evaluation of intervention effects. These efforts can support healthy aging and contribute to broader public health goals.

## Conclusion

Activity tracker-based interventions can be effective in promoting PA in older adults, particularly for increasing step counts and intensity-related PA outcomes, such as LPA and MVPA, in the short- and medium-term. Significant improvements were observed in activity tracker interventions that also incorporated multiple BCTs such as goal setting, self-monitoring, and feedback on behavior. However, their long-term efficacy and impact on reducing SB remain inconsistent. Moreover, unsuccessful interventions applied these BCTs too. Tailored interventions that address age-specific needs and sustained engagement are crucial. Future research should explore strategies to reduce SB independent of PA and address the optimal combination of BCTs. Overall, activity trackers hold promise as a tool to support healthy aging, but sustained behavior change requires further investigation.

## Supplementary Information

Additional file 1. Full search strategy of the selected databases.

Additional file 2. Reporting of study funding and conflict of interest statements in included trials.

Additional file 3. References of included studies.

Additional file 4. References of excluded studies – Population.

Additional file 5. References of excluded studies – Intervention

Additional file 6. References of excluded studies – Outcome.

Additional file 7. References of excluded studies – Study design.

Additional file 8. Characteristics of ongoing studies.

Additional file 9. Characteristics of studies awaiting classification.

Additional file 10. Overview of Behavior Change Techniques (BCTs) applied in activity tracker interventions.

## Data Availability

The datasets supporting the conclusions of this article are included within the article and its additional files.

## Abbreviation

BCTs: Behavior change techniques
COI: Conflict of Interest
IPAQ: International Physical Activity Questionnaire
LPA: Light Physical Activity
MET: Metabolic equivalent of task
MPA: Moderate physical activity
MVPA: Moderate-to-vigorous physical activity
PA: Physical activity
PASE: Physical Activity Scale for the Elderly
RCTs: Randomized controlled trials
RoB 2: Risk of Bias 2 tool
SB: Sedentary behavior
SD: Standard deviation
VPA: Vigorous physical activity
WSQ: Workforce Sitting Questionnaire
