# Supplementary material for: Efficacy of activity tracker-based interventions and their behavioral components in promoting physical activity and reducing sedentary behavior in older adults: a systematic review of randomized controlled trials
Source: Eur Rev Aging Phys Act. 2026 Jan 12;23:5. doi: 10.1186/s11556-025-00396-5 (PMC12853638; doi:10.1186/s11556-025-00396-5)
Supplement: Supplementary file 1 — Additional file 1. Full search strategy of the selected databases. [file 11556_2025_396_MOESM1_ESM.docx]

**Additional file 1.** Full search strategy of the selected databases

**PubMed**

1. ("Aged[MeSH] OR "Aged, 80 and over"[MeSH] OR Elderly OR geriatric OR "older people" OR "older adults" OR senior* OR "Frail Elderly"[MeSH] OR "older age" OR "old age")
2. ("Fitness Trackers" [MeSH] OR "fitness track*" OR wearable* OR "wearable device" OR "wearable electronic device*" OR "Wearable Health Device*" OR "wearable activ*" OR "activity track*" OR "physical activity track*" OR pedometer* OR "step count*" OR "track* technolog*" OR "activity monitor*" OR Smartphone OR App OR "Health App" OR "Mobile Application*")
3. ("sedentary behavior"[MeSH] OR sedentariness OR sedentary OR walking[MeSH] OR step* OR "physical activ*" OR "physical inactiv*" OR walk* OR "sitting time")"
4. #1 AND #2 AND #3
5. randomized controlled trial [pt] OR controlled clinical trial [pt] OR randomized [tiab] OR placebo [tiab] OR drug therapy [sh] OR randomly [tiab] OR trial [tiab] OR groups [tiab]
6. animals [mh] NOT humans [mh]
7. #5 NOT #6
8. #4 AND #7
9. #8 NOT (child*[TI] OR adolescent*[TI] OR "Observational Study"[TI] OR "longitudinal study"[TI] OR non-randomized[TI] OR nonrandomized[TI] OR "Cohort Study"[TI] OR "systematic review" [TI] OR "scoping review"[TI] OR "literature review"[TI] OR "umbrella review"[TI] OR "case control study" [TI] OR metaanalys*[TI] OR "meta-analys*"[TI] OR "cross-sectional study"[TI] OR "cross sectional study"[TI] OR "case study"[TI] OR "case series"[TI] OR school[TI])

**SportDiscus (EBSCOhost)**

1. ((DE "OLDER people" OR DE "GERIATRICS")) OR TI ((Aged OR "Aged, 80 and over" OR Elderly OR geriatric OR "older people" OR "older adults" OR senior* OR "Frail Elderly" OR "older age" OR "old age")) OR AB ((Aged OR "Aged, 80 and over" OR Elderly OR geriatric OR "older people" OR "older adults" OR senior* OR "Frail Elderly" OR "older age" OR "old age"))
2. ((DE "PEDOMETERS" OR DE "ACCELEROMETERS" OR DE "SPEEDOMETERS")) OR TI (("Fitness Trackers" OR "fitness track*" OR wearable* OR "wearable device" OR "wearable electronic device*" OR "Wearable Health Device*" OR "wearable activ*" OR "activity track*" OR "physical activity track*" OR pedometer* OR "step count*" OR "track* technolog*" OR "activity monitor*" OR Smartphone OR App OR "Health App" OR "Mobile Application*")) OT AB (("Fitness Trackers" OR "fitness track*" OR wearable* OR "wearable device" OR "wearable electronic device*" OR "Wearable Health Device*" OR "wearable activ*" OR "activity track*" OR "physical activity track*" OR pedometer* OR "step count*" OR "track* technolog*" OR "activity monitor*" OR Smartphone OR App OR "Health App" OR "Mobile Application*"))
3. ((DE "SEDENTARY lifestyles" OR DE "LIFESTYLES" OR DE "SEDENTARY lifestyles" OR DE "SEDENTARY people" OR DE "SEDENTARY women" OR DE "WALKING" OR DE "PHYSICAL activity" OR DE "PHYSICAL fitness")) OR TI (("sedentary behavior" OR sedentariness OR sedentary OR walking OR step* OR "physical activ*" OR "physical inactiv*" OR walk* OR "sitting time")) OR AB (("sedentary behavior" OR sedentariness OR sedentary OR walking OR step* OR "physical activ*" OR "physical inactiv*" OR walk* OR "sitting time"))
4. S1 AND S2 AND S3
5. S4 NOT ((child* OR adolescent* OR "Observational Study" OR "longitudinal study" OR non-randomized OR nonrandomized OR "Cohort Study" OR "systematic review" OR "scoping review" OR "literature review" OR "umbrella review" OR "case control study" OR metaanalys* OR "meta-analys*" OR "cross-sectional study" OR "cross sectional study" OR "case study" OR "case series" OR school))

**Cochrane Central Register of Controlled Trials (CENTRAL)**

1. MeSH descriptor: [Aged] explode all trees
2. MeSH descriptor: [Aged, 80 and over] explode all trees
3. MeSH descriptor: [Frail Elderly] explode all trees
4. (Elderly OR geriatric OR "older people" OR "older adults" OR senior* OR "older age" OR "old age"):ti,ab,kw
5. #1 OR #2 OR #3 OR #4
6. MeSH descriptor: [Fitness Trackers] explode all trees
7. MeSH descriptor: [Wearable Electronic Devices] explode all trees
8. MeSH descriptor: [Smartphone] explode all trees
9. MeSH descriptor: [Mobile Applications] explode all trees
10. ("fitness track*" OR wearable* OR "wearable device" OR "Wearable Health Device*" OR "wearable activ*" OR "activity track*" OR "physical activity track*" OR pedometer* OR "step count*" OR "track* technolog*" OR "activity monitor*" OR App OR "Health App"):ti,ab,kw
11. #6 OR #7 OR #8 OR #9 OR #10
12. MeSH descriptor: [Sedentary Behavior] explode all trees
13. MeSH descriptor: [Walking] explode all trees
14. (sedentariness OR sedentary OR step* OR "physical activ*" OR "physical inactiv*" OR walk* OR "sitting time"):ti,ab,kw
15. #12 OR #13 OR #14
16. #5 AND #11 AND #15
17. (child* OR adolescent* OR "Observational Study" OR "longitudinal study" OR non-randomized OR nonrandomized OR "Cohort Study" OR "systematic review" OR "scoping review" OR "literature review" OR "umbrella review" OR "case control study" OR metaanalys* OR "meta-analys*" OR "cross-sectional study" OR "cross sectional study" OR "case study" OR "case series" OR school):ti
18. #16 NOT #17

**Web of Science (Core collection)**

1. TS=(Aged Or "Aged, 80 and over" Or Elderly Or geriatric Or "older people"Or "older adults"Or senior* Or "Frail Elderly" Or "older age" Or "old age")
2. TS=(("Fitness Trackers" Or "fitness track*" Or wearable* Or "wearable device" Or "wearable electronic device*" Or "Wearable Health Device*" Or "wearable activ*" Or "activity track*" Or "physical activity track*" Or pedometer* Or "step count*" Or "track* technolog*" Or "activity monitor*" Or Smartphone Or App Or "Health App" Or "Mobile Application*"))
3. TS=(("sedentary behavior" Or sedentariness Or sedentary Or walking Or step* Or "physical activ*" Or "physical inactiv*" Or walk* Or "sitting time"))
4. #1 AND #2 AND #3
5. TI=((child* Or adolescent* Or "Observational Study" Or "longitudinal study" Or non-randomized Or nonrandomized Or "Cohort Study" Or "systematic review" Or "scoping review" Or "literature review" Or "umbrella review" Or "case control study" Or metaanalys* Or "meta-analys*" Or "cross-sectional study" Or "cross sectional study" Or "case study" Or "case series" Or school))
6. TS=(randomised OR randomized OR randomisation OR randomisation OR placebo* OR (random* AND (allocat* OR assign*)) OR (blind* AND (single OR double OR treble OR triple)))
7. #4 AND #6
8. #7 NOT #5
