# Supplementary material for: Efficacy of activity tracker-based interventions and their behavioral components in promoting physical activity and reducing sedentary behavior in older adults: a systematic review of randomized controlled trials
Source: Eur Rev Aging Phys Act. 2026 Jan 12;23:5. doi: 10.1186/s11556-025-00396-5 (PMC12853638; doi:10.1186/s11556-025-00396-5)
Supplement: Supplementary file 2 — Additional file 2. Reporting of study funding and conflict of interest statements in included trials. [file 11556_2025_396_MOESM2_ESM.docx]

**Additional file 2.** Reporting of study funding and conflict of interest statements in included trials

| **Author** | **Year** | **COI** | **Funding** |
| --- | --- | --- | --- |
| Alley et al. | 2022 | None | ‘SJA (102609) and SS (101240) are, and CV was (100427), supported by a fellowship from the National Heart Foundation of Australia. SS (1125586) and MJD (1141606) are supported by a fellowship from the National Health and Medical Research Council. The project was partially supported by a project grant from Central Queensland University and CV’s fellowship from the National Heart Foundation of Australia (100427). These funding agencies had no role in the design of the study; collection, analysis, and interpretation of data; or in writing and submitting the manuscript.’ |
| Bouchard et al. | 2013 | None | NR |
| Brickwood et al. | 2021 | None | ‘This research was supported by the internal funding provided by the University of Tasmania. In-kind support was provided by Primary Health Tasmania during the recruitment and data collection phase of this research. AW was supported by a Medical Research Future Fund Translating Research into Practice Fellowship.’ |
| Croteau et al. | 2004 | NR | ‘Research was supported by grants from University of Southern Maine College of Nursing and Health Professions, Payson Funds.’ |
| Croteau et al. | 2007 | NR | ‘This study was supported by a research grant from the  College of Nursing and Health Professions at the University  of Southern Maine.’ |
| Koizumi et al. | 2009 | NR | NR |
| Kolt et al. | 2012 | None | ‘The Health Research Council of New Zealand  (05/279R) and Sport and Recreation New Zealand funded this trial. The funders had no involvement in the research design, conduct, or interpretation of results.’ |
| Kwan et al. | 2020 | None | ‘This project is funded by the School of Nursing, The Hong Kong Polytechnic University (BE08).’ |
| Leskinen et al. | 2021 | None | ‘This work was supported by grants awarded by the Academy of Finland (grant no. 309526 to T. L. and grant no. 332030 to S. S.), the Juho Vainio Foundation (to T. L.), the Hospital District of Southwest Finland (to T. L.), and the Finnish Ministry of Education and Culture (to S. S.).’ |
| McLellan et al. | 2018 | None | ‘This project was funded by the Manitoba Medical Services  Foundation # 8-2013-06RS.’ |
| McMurdo et al. | 2010 | None | ‘The trial was funded by Scottish Executive Health Department Grant CZH/4/463. The funder had no role in the conduct, analysis or interpretation of the data.’ |
| Muellmann et al. | 2019 | None | ‘This study was funded by the German Federal Ministry of Education and Research (BMBF; project numbers 01EL1422A, 01EL1422C, 01EL1422E, 01EL1422F, 01EL1522I). The content of this article only reflects the authors' views and the funder is not liable for any use that  may be made of the information contained therein.’ |
| Roberts et al. | 2019 | ‘Dr Elizabeth Jackson is a paid consultant for McKesson as  well as for UpToDate, Inc. and has served as an expert witness for DeBlase Brown Everly LLP, all relationships are modest. Dr Jackson also received research funding from NIH, Amgen for epidemiology, medication utilization. She also served at the editorial board for American Heart Association (AHA) and served as editor/consultant for American College of Cardiology. Dr Todd M Manini reports grants from NIH and AHA, during the conduct of the study and grants from NIH and Regeneron, outside the submitted work. The authors report no other conflicts of interest in this work.’ | ‘This research was supported by the American Heart Association (16IRG27250237), National Institute on Aging (2P30AG028740), the National Center for Medical Rehabilitation Research (1P2CHD086851), and the University of Alabama at Birmingham Center for Exercise  Medicine.’ |
| Rowley et al. | 2019 | None | ‘This study was funded by a grant through the National Institute on Aging (5K01AG025962).’ |
| Slaght et al. | 2017 | NR | NR |
| Sudgen et al. | 2008 | None | ‘This study was funded by the Chief Scientist Office, Scottish Executive Health Department grant number CZH/4/310. The design, conduct and analysis of the study were completely independent of the funder.’ |
| Thomas et al. | 2012 | None | ‘The study was funded by the Hong Kong Government Health and Health Services Research Fund (HHSRF# 01030681).’ |
| Yamada et al. | 2012 | None | ‘Sponsor’s Role: None’ |

COI Conflict of Interest

None No COI or no funding received

NR Not reported
