# Supplementary material for: Efficacy of activity tracker-based interventions and their behavioral components in promoting physical activity and reducing sedentary behavior in older adults: a systematic review of randomized controlled trials
Source: Eur Rev Aging Phys Act. 2026 Jan 12;23:5. doi: 10.1186/s11556-025-00396-5 (PMC12853638; doi:10.1186/s11556-025-00396-5)
Supplement: Supplementary file 3 — Additional file 3. References of included studies. [file 11556_2025_396_MOESM3_ESM.docx]

# Additional file 3. References of included studies

**Alley et al. (2022)**

Alley, Stephanie; van Uffelen, Jannique Gz; Schoeppe, Stephanie; Parkinson, Lynne; Hunt, Susan; Power, Deborah et al. (2019): Efficacy of a computer-tailored web-based physical activity intervention using Fitbits for older adults: a randomised controlled trial protocol. In: *BMJ OPEN* 9 (12), e033305. DOI: 10.1136/bmjopen-2019-033305.

Alley, Stephanie J.; van Uffelen, Jannique; Schoeppe, Stephanie; Parkinson, Lynne; Hunt, Susan; Power, Deborah et al. (2022): The Effectiveness of a Computer-Tailored Web-Based Physical Activity Intervention Using Fitbit Activity Trackers in Older Adults (Active for Life): Randomized Controlled Trial. In: *JOURNAL OF MEDICAL INTERNET RESEARCH* 24 (5), e31352. DOI: 10.2196/31352.

**Bouchard et al. (2013)**

Bouchard, Danielle R.; Langlois, Marie-France; Boisvert-Vigneault, Katherine; Farand, Paul; Paulin, Mathieu; Baillargeon, Jean-Patrice (2013): Pilot study: can older inactive adults learn how to reach the required intensity of physical activity guideline? In: *CLINICAL INTERVENTIONS IN AGING* 8, S. 501–508. DOI: 10.2147/cia.s42224.

**Brickwood et al. (2021)**

Brickwood, Katie-Jane; Ahuja, Kiran D. K.; Watson, Greig; O’Brien, Jane A.; Williams, Andrew D. (2021): Effects of Activity Tracker Use With Health Professional Support or Telephone Counseling on Maintenance of Physical Activity and Health Outcomes in Older Adults: Randomized Controlled Trial. In: *JMIR MHEALTH AND UHEALTH* 9 (1), e18686. DOI: 10.2196/18686.

ACTRN12615001104549

**Croteau et al. (2004)**

Croteau, K. A.; Richeson, N. E.; Vines, S. W.; Jones, D. B. (2004): Effects of a pedometer-based physical activity program on older adults’ mobility-related self-efficacy and physical performance. In: *Activities, Adaptation & Aging* 28 (2), S. 19–33.

**Croteau et al. (2007)**

Croteau, Karen A.; Richeson, Nancy E.; Farmer, Bonnie C.; Jones, David B. (2007): Effect of a pedometer-based intervention on daily step counts of community-dwelling older adults. In: *RESEARCH QUARTERLY FOR EXERCISE AND SPORT* 78 (5), S. 401–406. DOI: 10.1080/02701367.2007.10599439.

**Koizumi et al. (2009)**

Koizumi, D.; Rogers, N. L.; Rogers, M. E.; Islam, M. M.; Kusunoki, M.; Takeshima, N. (2009): Efficacy of an Accelerometer-Guided Physical Activity Intervention in Community-Dwelling Older Women. In: *JOURNAL OF PHYSICAL ACTIVITY & HEALTH* 6 (4), S. 467–474. DOI: 10.1123/jpah.6.4.467.

**Kolt et al. (2012)**

ACTRN12606000023550 (2006): Healthy Steps: a trial of pedometer-based Green Prescription for older adults. In: *http://www.who.int/trialsearch/Trial2.aspx?TrialID=ACTRN12606000023550*.

Kolt, G.; Schofield, G. M.; Kerse, N.; Garrett, N.; Ashton, T. (2011): Healthy steps trial: effectiveness of a pedometer-based green prescription for low-active older adults in primary care. In: *Physiotherapy (united kingdom)* 97, eS630‐. DOI: 10.1016/j.physio.2011.04.002.

Kolt, Gregory S.; Schofield, Grant M.; Kerse, Ngaire; Garrett, Nicholas; Ashton, Toni; Patel, Asmita (2012): Healthy Steps trial: pedometer-based advice and physical activity for low-active older adults. In: *Annals of family medicine* 10 (3), S. 206–212. DOI: 10.1370/afm.1345.

Kolt, Gregory S.; Schofield, Grant M.; Kerse, Ngaire; Garrett, Nicholas; Schluter, Philip J.; Ashton, Toni; Patel, Asmita (2009): The healthy steps study: a randomized controlled trial of a pedometer-based green prescription for older adults. Trial protocol. In: *BMC PUBLIC HEALTH* 9, S. 404. DOI: 10.1186/1471-2458-9-404.

Patel, A.; Schofield, G.; Kolt, G. (2009): The effect of pedometer and time-based physical activity interventions on depressive symptomatology in low-active older adults. In: *Journal of Science & Medicine in Sport* 12, S16‐S16.

Patel, Asmita; Keogh, Justin W. L.; Kolt, Gregory S.; Schofield, Grant M. (2013): The long-term effects of a primary care physical activity intervention on mental health in low-active, community-dwelling older adults. In: *Aging & mental health* 17 (6), S. 766–772. DOI: 10.1080/13607863.2013.781118.

Patel, Asmita; Schofield, Grant M.; Kolt, Gregory S.; Keogh, Justin W. L. (2020): Older adults’ evaluations of the standard and modified pedometer-based Green Prescription. In: *Journal of primary health care* 12 (1), S. 41–48. DOI: 10.1071/hc19007.

**Kwan et al. (2020)**

Kwan, R. Y. C.; Lee, D.; Lee, P. H.; Tse, M.; Cheung, D. S. K.; Thiamwong, L.; Choi, K. S. (2020): Effects of an mHealth Brisk Walking Intervention on Increasing Physical Activity in Older People With Cognitive Frailty: Pilot Randomized Controlled Trial. In: *JMIR MHEALTH AND UHEALTH* 8 (7). DOI: 10.2196/16596.

HKUCTR-2283

**Leskinen et al. (2021)**

NCT03320746

Leskinen, Tuija; Suorsa, Kristin; Tuominen, Miika; Pulakka, Anna; Pentti, Jaana; Löyttyniemi, Eliisa et al. (2021): The Effect of Consumer-based Activity Tracker Intervention on Physical Activity among Recent Retirees-An RCT Study. In: *Medicine and science in sports and exercise* 53 (8), S. 1756–1765. DOI: 10.1249/mss.0000000000002627.

Leskinen, Tuija; Suorsa, Kristin; Heinonen, I.H.; Löyttyniemi, Eliisa; Pentti, Jaana; Vahtera, J.; et al. (2021): The Effect of Commercial Activity Tracker Based Physical Activity Intervention on Body Composition and Cardiometabolic Health Among Recent Retirees. In: *Frontiers in Aging* 2021 (2)

Pasanen, Jesse; Leskinen, Tuija; Suorsa, Kristin; Pulakka, Anna; Virta, Joni; Auranen, Kari; Stenholm, Sari (2022): Effects of physical activity intervention on 24-h movement behaviors: a compositional data analysis. In: *SCIENTIFIC REPORTS* 12 (1), S. 8712. DOI: 10.1038/s41598-022-12715-2.

Suorsa, Kristin; Leskinen, Tuija; Pulakka, Anna; Pentti, Jaana; Löyttyniemi, Eliisa; Heinonen, Ilkka et al. (2022): The Effect of a Consumer-Based Activity Tracker Intervention on Accelerometer-Measured Sedentary Time Among Retirees: A Randomized Controlled REACT Trial. In: *The journals of gerontology. Series A, Biological sciences and medical sciences* 77 (3), S. 579–587. DOI: 10.1093/gerona/glab107.

Tuominen, Miika; Suorsa, Kristin; Pentti, Jaana; Koski, Pasi; Stenholm, Sari; Leskinen, Tuija (2021): The Impact of a 12-Month Activity Tracker Intervention on Activity Behavior Across Body Mass Index Subgroups Among Recent Retirees: Post Hoc Analysis of a Randomized Controlled Trial. In: *JOURNAL OF PHYSICAL ACTIVITY & HEALTH* 18 (12), S. 1563–1569. DOI: 10.1123/jpah.2021-0352.

**McLellan et al. (2018)**

McLellan, A. G.; Slaght, Jana; Craig, C. M.; Mayo, A.; Sénéchal, M.; Bouchard, Danielle R. (2018): Can older adults improve the identification of moderate intensity using walking cadence? In: *AGING CLINICAL AND EXPERIMENTAL RESEARCH* 30 (1), S. 89–92. DOI: 10.1007/s40520-017-0746-3.

**McMurdo et al. (2010)**

ISRCTN26786857

McMurdo, Marion E. T.; Sugden, Jacqui; Argo, Ishbel; Boyle, Paul; Johnston, Derek W.; Sniehotta, Falko F.; Donnan, Peter T. (2010): Do pedometers increase physical activity in sedentary older women? A randomized controlled trial. In: *JOURNAL OF THE AMERICAN GERIATRICS SOCIETY* 58 (11), S. 2099–2106. DOI: 10.1111/j.1532-5415.2010.03127.x.

**Muellmann et al. (2019)**

DRKS00010052

Muellmann, Saskia; Bragina, Inna; Voelcker-Rehage, Claudia; Rost, Eric; Lippke, Sonia; Meyer, Jochen et al. (2017): Development and evaluation of two web-based interventions for the promotion of physical activity in older adults: study protocol for a community-based controlled intervention trial. In: *BMC PUBLIC HEALTH* 17 (1), S. 512. DOI: 10.1186/s12889-017-4446-x.

Muellmann, Saskia; Buck, Christoph; Voelcker-Rehage, Claudia; Bragina, Inna; Lippke, Sonia; Meyer, Jochen et al. (2019): Effects of two web-based interventions promoting physical activity among older adults compared to a delayed intervention control group in Northwestern Germany: Results of the PROMOTE community-based intervention trial. In: *PREVENTIVE MEDICINE REPORTS* 15, S. 100958. DOI: 10.1016/j.pmedr.2019.100958.

Ratz, Tiara; Lippke, Sonia; Muellmann, Saskia; Peters, Manuela; Pischke, Claudia R.; Meyer, Jochen et al. (2020): Effects of Two Web-Based Interventions and Mediating Mechanisms on Stage of Change Regarding Physical Activity in Older Adults. In: *APPLIED PSYCHOLOGY-HEALTH AND WELL BEING* 12 (1), S. 77–100. DOI: 10.1111/aphw.12174.

**Roberts et al. (2019)**

NCT02632487 (2015): Wearable Technology to Reduce Sedentary Behavior and CVD Risk in Older Adults. In: *https://clinicaltrials.gov/show/NCT02632487*.

Roberts, L. M.; Jaeger, B. C.; Baptista, L. C.; Harper, S. A.; Gardner, A. K.; Jackson, E. A. et al. (2019): Wearable Technology To Reduce Sedentary Behavior And CVD Risk In Older Adults: a Pilot Randomized Clinical Trial. In: *CLINICAL INTERVENTIONS IN AGING* 14, S. 1817–1828. DOI: 10.2147/cia.s222655.

Krehbiel, Lisa M.; Layne, Andrew S.; Sandesara, Bhanuprasad; Manini, Todd M.; Anton, Stephen D.; Buford, Thomas W. (2017): Wearable technology to reduce sedentary behavior and CVD risk in older adults: design of a randomized controlled trial. In: *CONTEMPORARY CLINICAL TRIALS COMMUNICATIONS* 6, S. 122–126. DOI: 10.1016/j.conctc.2017.04.003.

**Rowley et al. (2019)**

Rowley, Taylor W.; Lenz, Elizabeth K.; Swartz, Ann M.; Miller, Nora E.; Maeda, Hotaka; Strath, Scott J. (2019): Efficacy of an Individually Tailored, Internet-Mediated Physical Activity Intervention in Older Adults: A Randomized Controlled Trial. In: *Journal of applied gerontology : the official journal of the Southern Gerontological Society* 38 (7), S. 1011–1022. DOI: 10.1177/0733464817735396.

**Slaght et al. (2017)**

Slaght, Jana; Sénéchal, Martin; Bouchard, Danielle R. (2017): Impact of Walking Cadence Prescription to Reach the Global Physical Activity Recommendations in Older Adults. In: *Journal of Aging & Physical Activity* 25 (4), S. 604–611.

**Sugden et al. (2008)**

Sugden, Jacqui A.; Sniehotta, Falko F.; Donnan, Peter T.; Boyle, Paul; Johnston, Derek W.; McMurdo, Marion E. T. (2008): The feasibility of using pedometers and brief advice to increase activity in sedentary older women‐a pilot study. In: *BMC health services research* 8, S. 169. DOI: 10.1186/1472-6963-8-169.

**Thomas et al. (2012)**

HKUCTR-346

Thomas, G. N.; Macfarlane, D. J.; Guo, B. L.; Cheung, B. M. Y.; McGhee, S. M.; Chou, K. L. et al. (2012): Health Promotion in Older Chinese: A 12-Month Cluster Randomized Controlled Trial of Pedometry and "Peer Support. In: *MEDICINE & SCIENCE IN SPORTS & EXERCISE* 44 (6), S. 1157–1166. DOI: 10.1249/MSS.0b013e318244314a.

**Yamada et al. (2012)**

Yamada, M.; Mori, S.; Nishiguchi, S.; Kajiwara, Y.; Yoshimura, K.; Sonoda, T. et al. (2012): Pedometer-Based Behavioral Change Program Can Improve Dependency in Sedentary Older Adults: A Randomized Controlled Trial. In: *The Journal of frailty & aging* 1 (1), S. 39–44. DOI: 10.14283/jfa.2012.7.
