# Supplementary material for: Efficacy of activity tracker-based interventions and their behavioral components in promoting physical activity and reducing sedentary behavior in older adults: a systematic review of randomized controlled trials
Source: Eur Rev Aging Phys Act. 2026 Jan 12;23:5. doi: 10.1186/s11556-025-00396-5 (PMC12853638; doi:10.1186/s11556-025-00396-5)
Supplement: Supplementary file 4 — Additional file 4. References of excluded studies – Population. [file 11556_2025_396_MOESM4_ESM.docx]

# Additional file 4. References of excluded studies - Population Reason for exclusion: Population

1. Abedi, P.; Nikkhah, P.; Najar, S. (2015): Effect of pedometer-based walking on depression, anxiety and insomnia among postmenopausal women. In: *Climacteric : the journal of the International Menopause Society* 18 (6), S. 841–845. DOI: 10.3109/13697137.2015.1065246.
2. Arbour, K. P.; Martin Ginis, K. A. (2008): Improving body image one step at a time: greater pedometer step counts produce greater body image improvements. In: *Body image* 5 (4), S. 331–336. DOI: 10.1016/j.bodyim.2008.05.003.
3. Armit, C. M.; Brown, W. J.; Ritchie, C. B.; Trost, S. G. (2005): Promoting physical activity to older adults: a preliminary evaluation of three general practice-based strategies. In: *JOURNAL OF SCIENCE AND MEDICINE IN SPORT* 8 (4), S. 446–450. DOI: 10.1016/s1440-2440(05)80060-x.
4. Armit, Christine M.; Brown, Wendy J.; Marshall, Alison L.; Ritchie, Carrie B.; Trost, Stewart G.; Green, Anita; Bauman, Adrian E. (2009): Randomized trial of three strategies to promote physical activity in general practice. In: *PREVENTIVE MEDICINE* 48 (2), S. 156–163. DOI: 10.1016/j.ypmed.2008.11.009.
5. Ashe, Maureen C.; Winters, Meghan; Hoppmann, Christiane A.; Dawes, Martin G.; Gardiner, Paul A.; Giangregorio, Lora M. et al. (2015): Not just another walking program": Everyday Activity Supports You (EASY) model-a randomized pilot study for a parallel randomized controlled trial. In: *PILOT AND FEASIBILITY STUDIES* 1, S. 4. DOI: 10.1186/2055-5784-1-4.
6. Buman, Matthew P.; Giacobbi, Peter R.; Dzierzewski, Joseph M.; Morgan, Adrienne Aiken; McCrae, Christina S.; Roberts, Beverly L.; Marsiske, Michael (2011): Peer Volunteers Improve Long-Term Maintenance of Physical Activity With Older Adults: A Randomized Controlled Trial. In: *JOURNAL OF PHYSICAL ACTIVITY & HEALTH* 8 (s2), S257‐S266. DOI: 10.1123/jpah.8.s2.s257.
7. Cadmus-Bertram Randomized Trial of a Fitbit-Based Physical Activity Intervention for Women Am J Prev Med 2015
8. Choi, JiWon; Shin, Nah-Mee; Cooper, Bruce; Jih, Jane; Janice, Tsoh (2021): A Pilot Study to Promote Active Living among Physically Inactive Korean American Women. In: *Journal of community health nursing* 38 (1), S. 24–37. DOI: 10.1080/07370016.2021.1869420.
9. Compernolle. Effectiveness of a web-based, computer-tailored, pedometer-based physical activity intervention for adults: a cluster randomized controlled trial. J Med Internet Res. 2015 Feb 9;17(2):e38. doi: 10.2196/jmir.3402
10. Darker, C. D.; French, D. P.; Eves, F. F.; Sniehotta, F. F. (2010): An intervention to promote walking amongst the general population based on an ’extended’ theory of planned behaviour: a waiting list randomised controlled trial. In: *PSYCHOLOGY & HEALTH* 25 (1), S. 71–88. DOI: 10.1080/08870440902893716.
11. De Cocker, K. A.; de Im Bourdeaudhuij; Brown, W. J.; Cardon, G. M. (2011): Four-year follow-up of the community intervention ’10 000 steps Ghent’. In: *HEALTH EDUCATION RESEARCH* 26 (2), S. 372–380. DOI: 10.1093/her/cyr015.
12. De Cocker, Katrien; Spittaels, Heleen; Cardon, Greet; Bourdeaudhuij, Ilse de; Vandelanotte, Corneel (2012): Web-based, computer-tailored, pedometer-based physical activity advice: development, dissemination through general practice, acceptability, and preliminary efficacy in a randomized controlled trial. In: *JOURNAL OF MEDICAL INTERNET RESEARCH* 14 (2), e53. DOI: 10.2196/jmir.1959.
13. Duncan, Mitch J.; Vandelanotte, Corneel; Trost, Stewart G.; Rebar, Amanda L.; Rogers, Naomi; Burton, Nicola W. et al. (2016): Balanced: a randomised trial examining the efficacy of two self-monitoring methods for an app-based multi-behaviour intervention to improve physical activity, sitting and sleep in adults. In: *BMC PUBLIC HEALTH* 16, S. 670. DOI: 10.1186/s12889-016-3256-x.
14. Ellingson, Laura D.; Lansing, Jeni E.; DeShaw, Kathryn J.; Peyer, Karissa L.; Bai, Yang; Perez, Maria et al. (2019): Evaluating Motivational Interviewing and Habit Formation to Enhance the Effect of Activity Trackers on Healthy Adults’ Activity Levels: Randomized Intervention. In: *JMIR MHEALTH AND UHEALTH* 7 (2), e10988. DOI: 10.2196/10988.
15. Ewald, Ben; Stacey, Fiona; Johnson, Natalie; Plotnikoff, Ronald C.; Holliday, Elizabeth; Brown, Wendy; James, Erica L. (2018): Physical activity coaching by Australian Exercise Physiologists is cost effective for patients referred from general practice. In: *AUSTRALIAN AND NEW ZEALAND JOURNAL OF PUBLIC HEALTH* 42 (1), S. 12–15. DOI: 10.1111/1753-6405.12733.
16. Fitzsimons, Claire F.; Baker, Graham; Gray, Stuart R.; Nimmo, Myra A.; Mutrie, Nanette (2012): Does physical activity counselling enhance the effects of a pedometer-based intervention over the long-term: 12-month findings from the Walking for Wellbeing in the west study. In: *BMC PUBLIC HEALTH* 12, S. 206. DOI: 10.1186/1471-2458-12-206.
17. Fitzsimons, Claire F.; Baker, Graham; Wright, Annemarie; Nimmo, Myra A.; Ward Thompson, Catharine; Lowry, Ruth et al. (2008): The ’Walking for Wellbeing in the West’ randomised controlled trial of a pedometer-based walking programme in combination with physical activity consultation with 12 month follow-up: rationale and study design. In: *BMC PUBLIC HEALTH* 8, S. 259. DOI: 10.1186/1471-2458-8-259.
18. French, D. P.; Williams, S. L.; Michie, S.; Taylor, C.; Szczepura, A.; Stallard, N.; Dale, J. (2011): A cluster randomised controlled trial of the efficacy of a brief walking intervention delivered in primary care: Study protocol. In: *BMC FAMILY PRACTICE* 12. DOI: 10.1186/1471-2296-12-56.
19. Gill, D. P.; Blunt, W.; Boa Sorte Silva, N. C.; Stiller-Moldovan, C.; Zou, G. Y.; Petrella, R. J. (2019): The HealtheSteps™ lifestyle prescription program to improve physical activity and modifiable risk factors for chronic disease: a pragmatic randomized controlled trial. In: *BMC PUBLIC HEALTH* 19 (1), S. 841. DOI: 10.1186/s12889-019-7141-2.
20. Glynn Effectiveness of a smartphone application to promote physical activity in primary care: the SMART MOVE randomised controlled trial Br J Gen Pract 2014
21. Gur, F.; Gur, G. C.; Ayan, V. (2020): The Effect of the ERVE Smartphone App on Physical Activity, Quality of Life, Self-Efficacy, and Exercise Motivation for Inactive People: A Randomized Controlled Trial. In: *EUROPEAN JOURNAL OF INTEGRATIVE MEDICINE* 39. DOI: 10.1016/j.eujim.2020.101198.
22. Kappen, D. L.; Mirza-Babaei, P.; Le Nacke (2020): Technology Facilitates Physical Activity Through Gamification: A Thematic Analysis of an 8-Week Study. In: *FRONTIERS IN COMPUTER SCIENCE* 2. DOI: 10.3389/fcomp.2020.530309.
23. Khunti, Kamlesh; Griffin, Simon; Brennan, Alan; Dallosso, Helen; Davies, Melanie J.; Eborall, Helen C. et al. (2021): Promoting physical activity in a multi-ethnic population at high risk of diabetes: the 48-month PROPELS randomised controlled trial. In: *BMC medicine* 19 (1), S. 130. DOI: 10.1186/s12916-021-01997-4.
24. King, Abby C.; Hekler, Eric B.; Grieco, Lauren A.; Winter, Sandra J.; Sheats, Jylana L.; Buman, Matthew P. et al. (2016): Effects of Three Motivationally Targeted Mobile Device Applications on Initial Physical Activity and Sedentary Behavior Change in Midlife and Older Adults: A Randomized Trial. In: *PLOS ONE* 11 (6), e0156370. DOI: 10.1371/journal.pone.0156370.
25. Kolt, Gregory S.; Rosenkranz, Richard R.; Vandelanotte, Corneel; Caperchione, Cristina M.; Maeder, Anthony J.; Tague, Rhys et al. (2017): Using Web 2.0 applications to promote health-related physical activity: findings from the WALK 2.0 randomised controlled trial. In: *BRITISH JOURNAL OF SPORTS MEDICINE* 51 (19), S. 1433–1440. DOI: 10.1136/bjsports-2016-096890.
26. Kovelis Using Web 2.0 applications to promote health-related physical activity: findings from the WALK 2.0 randomised controlled trial Br J Sports Med 2017
27. Kriska, A. M.; Bayles, C.; Cauley, J. A.; LaPorte, R. E.; Sandler, R. B.; Pambianco, G. (1986): A randomized exercise trial in older women: increased activity over two years and the factors associated with compliance. In: *Medicine and science in sports and exercise* 18 (5), S. 557–562.
28. Lane, Aoife; Murphy, Niamh; Bauman, Adrian (2015): An effort to ’leverage’ the effect of participation in a mass event on physical activity. In: *Health promotion international* 30 (3), S. 542–551. DOI: 10.1093/heapro/dat077.
29. Lara, Jose; O’Brien, Nicola; Godfrey, Alan; Heaven, Ben; Evans, Elizabeth H.; Lloyd, Scott et al. (2016): Pilot Randomised Controlled Trial of a Web-Based Intervention to Promote Healthy Eating, Physical Activity and Meaningful Social Connections Compared with Usual Care Control in People of Retirement Age Recruited from Workplaces. In: *PLOS ONE* 11 (7), e0159703. DOI: 10.1371/journal.pone.0159703.
30. Lee, Tony Szu-Hsien; Hung, Chia-Chun; Lin, Chao-Kuang; Chiang, Hui-Hsun (2019): Controlled randomized trial of walking exercise with positive education on cardiovascular fitness and happiness in retired older adults. In: *GERIATRICS & GERONTOLOGY INTERNATIONAL* 19 (9), S. 879–884. DOI: 10.1111/ggi.13733.
31. Lewis, Zakkoyya H.; Ottenbacher, Kenneth J.; Fisher, Steve R.; Jennings, Kristofer; Brown, Arleen F.; Swartz, Maria C. et al. (2017): The feasibility and RE-AIM evaluation of the TAME health pilot study. In: *INTERNATIONAL JOURNAL OF BEHAVIORAL NUTRITION AND PHYSICAL ACTIVITY* 14 (1), S. 106. DOI: 10.1186/s12966-017-0560-5.
32. Lewis, Zakkoyya H.; Ottenbacher, Kenneth J.; Fisher, Steve R.; Jennings, Kristofer; Brown, Arleen F.; Swartz, Maria C. et al. (2020): Effect of Electronic Activity Monitors and Pedometers on Health: Results from the TAME Health Pilot Randomized Pragmatic Trial. In: *INTERNATIONAL JOURNAL OF ENVIRONMENTAL RESEARCH AND PUBLIC HEALTH* 17 (18). DOI: 10.3390/ijerph17186800.
33. Liu, Justina Y. W.; Kwan, Rick Y. C.; Yin, Yue-Heng; Lee, Paul H.; Siu, Judy Yuen-Man; Bai, Xue (2021): Enhancing the Physical Activity Levels of Frail Older Adults with a Wearable Activity Tracker-Based Exercise Intervention: A Pilot Cluster Randomized Controlled Trial. In: *INTERNATIONAL JOURNAL OF ENVIRONMENTAL RESEARCH AND PUBLIC HEALTH* 18 (19). DOI: 10.3390/ijerph181910344.
34. Lyons Feasibility and Acceptability of a Wearable Technology Physical Activity Intervention With Telephone Counseling for Mid-Aged and Older Adults: A Randomized Controlled Pilot Trial JMIR Mhealth Uhealth 2017
35. Malik, M. A.; Suboc, T.; Strath, S. J.; Wang, J.; Tanner, M. J.; Ying, R.; Widlansky, M. (2015): Impact of increasing physical activity over 12 weeks in previously sedentary older adults: a 1-year follow up. In: *JOURNAL OF THE AMERICAN COLLEGE OF CARDIOLOGY* 65 (10 SUPPL. 1), A1451.
36. Mann. A pilot randomized trial of technology-assisted goal setting to improve physical activity among primary care patients with prediabetes. Prev Med Rep. 2016 May 21:4:107-12. doi: 10.1016/j.pmedr.2016.05.012.
37. Marshall, Alison L. (2007): Should all steps count when using a pedometer as a measure of physical activity in older adults? In: *JOURNAL OF PHYSICAL ACTIVITY & HEALTH* 4 (3), S. 305–314. DOI: 10.1123/jpah.4.3.305.
38. Moffitt, Robyn; Mohr, Philip (2015): The efficacy of a self-managed Acceptance and Commitment Therapy intervention DVD for physical activity initiation. In: *BRITISH JOURNAL OF HEALTH PSYCHOLOGY* 20 (1), S. 115–129. DOI: 10.1111/bjhp.12098.
39. Morais Pereira Simoes, M. D. S.; Barros Gonze, B. de; Leite Proenca, N.; Tonon Lauria, V.; Demarchi Silva Terra, V.; Da Costa Padovani, R.; Zuniga Dourado, V. (2019): Use of a smartphone app combined with gamification to increase the level of physical activity of adults and older adults: protocol of a sequential multiple assignment randomized trial. In: *TRIALS* 20 (1). DOI: 10.1186/s13063-019-3879-1.
40. Murawski, B.; Plotnikoff, R. C.; Rayward, A. T.; Vandelanotte, C.; Brown, W. J.; Duncan, M. J. (2018): Randomised controlled trial using a theory-based m-health intervention to improve physical activity and sleep health in adults: the Synergy Study protocol. In: *BMJ OPEN* 8 (2). DOI: 10.1136/bmjopen-2017-018997.
41. Murawski, Beatrice; Plotnikoff, Ronald C.; Rayward, Anna T.; Oldmeadow, Christopher; Vandelanotte, Corneel; Brown, Wendy J.; Duncan, Mitch J. (2019): Efficacy of an m-Health Physical Activity and Sleep Health Intervention for Adults: A Randomized Waitlist-Controlled Trial. In: *AMERICAN JOURNAL OF PREVENTIVE MEDICINE* 57 (4), S. 503–514. DOI: 10.1016/j.amepre.2019.05.009.
42. NCT01212978 (2010): Overcoming Inactivity in Older Adults: impact on Vascular Homeostasis. In: *https://clinicaltrials.gov/show/NCT01212978*.
43. NCT01869348 (2013): IMPACT: inactivity Monitoring and Physical Activity Controlled Trial. In: *https://clinicaltrials.gov/show/NCT01869348*.
44. Norton, Lynda H.; Norton, Kevin I.; Lewis, Nicole R. (2015): Adherence, Compliance, and Health Risk Factor Changes following Short-Term Physical Activity Interventions. In: *BIOMED RESEARCH INTERNATIONAL* 2015, S. 929782. DOI: 10.1155/2015/929782.
45. Oppezzo, M. A.; Tremmel, J. A.; Kapphahn, K.; Desai, M.; Baiocchi, M.; Sanders, M.; Prochaska, J. J. (2021): Feasibility, preliminary efficacy, and accessibility of a twitter-based social support group vs Fitbit only to decrease sedentary behavior in women. In: *INTERNET INTERVENTIONS-THE APPLICATION OF INFORMATION TECHNOLOGY IN MENTAL AND BEHAVIOURAL HEALTH* 25. DOI: 10.1016/j.invent.2021.100426.
46. Patel, Mitesh S.; Polsky, Daniel; Kennedy, Edward H.; Small, Dylan S.; Evans, Chalanda N.; Rareshide, Charles A. L.; Volpp, Kevin G. (2020): Smartphones vs Wearable Devices for Remotely Monitoring Physical Activity After Hospital Discharge: A Secondary Analysis of a Randomized Clinical Trial. In: *JAMA NETWORK OPEN* 3 (2), e1920677. DOI: 10.1001/jamanetworkopen.2019.20677.
47. Pears A randomised controlled trial of three very brief interventions for physical activity in primary care BMC Public Health 2016
48. Pekmezi, Dorothy; Dunsiger, Shira; Gaskins, Ronnesia; Barbera, Brooke; Marquez, Becky; Neighbors, Charles; Marcus, Bess (2013): Feasibility and acceptability of using pedometers as an intervention tool for Latinas. In: *JOURNAL OF PHYSICAL ACTIVITY & HEALTH* 10 (3), S. 451–457. DOI: 10.1123/jpah.10.3.451.
49. Petersen, Christina Bjørk; Severin, Maria; Hansen, Andreas Wolff; Curtis, Tine; Grønbæk, Morten; Tolstrup, Janne Schurmann (2012): A population-based randomized controlled trial of the effect of combining a pedometer with an intervention toolkit on physical activity among individuals with low levels of physical activity or fitness. In: *PREVENTIVE MEDICINE* 54 (2), S. 125–130. DOI: 10.1016/j.ypmed.2011.12.012.
50. Petry, Nancy M.; Andrade, Leonardo F.; Barry, Danielle; Byrne, Shannon (2013): A randomized study of reinforcing ambulatory exercise in older adults. In: *PSYCHOLOGY AND AGING* 28 (4), S. 1164–1173. DOI: 10.1037/a0032563.
51. Rockette-Wagner, Bonny; Fischer, Gary S.; Kriska, Andrea M.; Conroy, Molly B.; Dunstan, David; Roumpz, Caroline; McTigue, Kathleen M. (2020): Efficacy of an Online Physical Activity Intervention Coordinated With Routine Clinical Care: Protocol for a Pilot Randomized Controlled Trial. In: *JMIR RESEARCH PROTOCOLS* 9 (11), e18891. DOI: 10.2196/18891.
52. Romero, Zasha; Kimbrough, Sandy (2019): The Impact of Moderate Physical Activity on Weight and Blood Pressure in an Elderly Mexican-American Female Population. In: *TAHPERD Journal* 87 (3), S. 8–12.
53. Rosenberg, D.E.; Kadokura, E.; Morris, M. E.; Renz, A.; Vilardaga, R. M. (2017): Application of N-of-1 Experiments to Test the Efficacy of Inactivity Alert Features in Fitness Trackers to Increase Breaks from Sitting in Older Adults. In: *METHODS OF INFORMATION IN MEDICINE* 56 (6), S. 427–436. DOI: 10.3414/me16-02-0043.
54. Sawchuk, Craig N.; Charles, Steve; Wen, Yang; Goldberg, Jack; Forquera, Ralph; Roy-Byrne, Peter; Buchwald, Dedra (2008): A randomized trial to increase physical activity among native elders. In: *PREVENTIVE MEDICINE* 47 (1), S. 89–94. DOI: 10.1016/j.ypmed.2008.03.011.
55. Sawchuk, Craig N.; Russo, Joan E.; Charles, Steve; Goldberg, Jack; Forquera, Ralph; Roy-Byrne, Peter; Buchwald, Dedra (2011): Does pedometer goal setting improve physical activity among Native elders? Results from a randomized pilot study. In: *American Indian and Alaska native mental health research (Online)* 18 (1), S. 23–41. DOI: 10.5820/aian.1801.2011.23.
56. Sidman, Cara L.; Corbin, Charles B.; Le Masurier, Guy (2004): Promoting physical activity among sedentary women using pedometers. In: *RESEARCH QUARTERLY FOR EXERCISE AND SPORT* 75 (2), S. 122–129.
57. Suboc, Tisha B.; Knabel, Daniel; Strath, Scott J.; Dharmashankar, Kodlipet; Coulliard, Allison; Malik, Mobin et al. (2016): Associations of Reducing Sedentary Time With Vascular Function and Insulin Sensitivity in Older Sedentary Adults. In: *AMERICAN JOURNAL OF HYPERTENSION* 29 (1), S. 46–53. DOI: 10.1093/ajh/hpv063.
58. Suboc, Tisha B.; Strath, Scott J.; Dharmashankar, Kodlipet; Coulliard, Allison; Miller, Nora; Wang, Jingli et al. (2014): Relative importance of step count, intensity, and duration on physical activity’s impact on vascular structure and function in previously sedentary older adults. In: *JOURNAL OF THE AMERICAN HEART ASSOCIATION* 3 (1), e000702. DOI: 10.1161/jaha.113.000702.
59. Suboc, Tisha B.; Strath, Scott J.; Dharmashankar, Kodlipet; Harmann, Leanne; Couillard, Allison; Malik, Mobin et al. (2014): The Impact of Moderate Intensity Physical Activity on Cardiac Structure and Performance in Older Sedentary Adults. In: *International journal of cardiology. Heart & vessels* 4, S. 19–24. DOI: 10.1016/j.ijchv.2014.08.007.
60. Tadayon, M.; Abedi, P.; Farshadbakht, F. (2016): Impact of pedometer-based walking on menopausal women’s sleep quality: a randomized controlled trial. In: *Climacteric : the journal of the International Menopause Society* 19 (4), S. 364–368. DOI: 10.3109/13697137.2015.1123240.
61. Tudor-Locke, Catrine; Schuna, John M.; Swift, Damon L.; Dragg, Amber T.; Davis, Allison B.; Martin, Corby K. et al. (2020): Evaluation of Step-Counting Interventions Differing on Intensity Messages. In: *JOURNAL OF PHYSICAL ACTIVITY & HEALTH* 17 (1), S. 21–28. DOI: 10.1123/jpah.2018-0439.
62. Tudor-Locke, Catrine; Swift, Damon L.; Schuna, John M.; Dragg, Amber T.; Davis, Allison B.; Martin, Corby K. et al. (2014): WalkMore: a randomized controlled trial of pedometer-based interventions differing on intensity messages. In: *BMC PUBLIC HEALTH* 14, S. 168. DOI: 10.1186/1471-2458-14-168.
63. van Hoye, Karen; Boen, Filip; Lefevre, Johan (2015): The Impact of Different Degrees of Feedback on Physical Activity Levels: A 4-Week Intervention Study. In: *INTERNATIONAL JOURNAL OF ENVIRONMENTAL RESEARCH AND PUBLIC HEALTH* 12 (6), S. 6561–6581. DOI: 10.3390/ijerph120606561.
64. Van Hoye Year-round effects of a four-week randomized controlled trial using different types of feedback on employees' physical activity BMC Public Health 2018
65. Vandelanotte, Corneel; Duncan, Mitch J.; Maher, Carol A.; Schoeppe, Stephanie; Rebar, Amanda L.; Power, Deborah A. et al. (2018): The Effectiveness of a Web-Based Computer-Tailored Physical Activity Intervention Using Fitbit Activity Trackers: Randomized Trial. In: *JOURNAL OF MEDICAL INTERNET RESEARCH* 20 (12), e11321. DOI: 10.2196/11321.
66. Warren Evaluation of different recruitment and randomisation methods in a trial of general practitioner-led interventions to increase physical activity: a randomised controlled feasibility study with factorial design Trials 2014
67. Yang, Ya-Ping; Wang, Chi-Jane; Wang, Jing-Jy; Lin, Che-Wei; Yang, Ya-Ting Carolyn; Wang, Jeen-Shing et al. (2017): The Effects of an Activity Promotion System on active living in overweight subjects with metabolic abnormalities. In: *Obesity research & clinical practice* 11 (6), S. 718–727. DOI: 10.1016/j.orcp.2017.06.002.
68. Yates Effectiveness of a pragmatic education program designed to promote walking activity in individuals with impaired glucose tolerance: a randomized controlled trial Diabetes Care 2009
69. Yates Walking Away from Type 2 diabetes: a cluster randomized controlled trial Diabet Med 2017
70. Zabatiero Comparison of two strategies using pedometers to counteract physical inactivity in smokers Nicotine Tob Res 2014
