# Supplementary material for: Efficacy of activity tracker-based interventions and their behavioral components in promoting physical activity and reducing sedentary behavior in older adults: a systematic review of randomized controlled trials
Source: Eur Rev Aging Phys Act. 2026 Jan 12;23:5. doi: 10.1186/s11556-025-00396-5 (PMC12853638; doi:10.1186/s11556-025-00396-5)
Supplement: Supplementary file 5 — Additional file 5. References of excluded studies – Intervention [file 11556_2025_396_MOESM5_ESM.docx]

# Additional file 5. References of excluded studies – Intervention

# Reason for exclusion: Intervention

1. Bickmore, Timothy W.; Silliman, Rebecca A.; Nelson, Kerrie; Cheng, Debbie M.; Winter, Michael; Henault, Lori; Paasche-Orlow, Michael K. (2013): A randomized controlled trial of an automated exercise coach for older adults. In: *JOURNAL OF THE AMERICAN GERIATRICS SOCIETY* 61 (10), S. 1676–1683. DOI: 10.1111/jgs.12449.
2. Burke, Linda; Jancey, Jonine; Howat, Peter; Lee, Andy; Kerr, Deborah; Shilton, Trevor et al. (2010): Physical activity and nutrition program for seniors (PANS): protocol of a randomized controlled trial. In: *BMC PUBLIC HEALTH* 10, S. 751. DOI: 10.1186/1471-2458-10-751.
3. Cai, X.; Qiu, S. H.; Luo, D.; Li, R. X.; Liu, C. Y.; Lu, Y. H. et al. (2022): Effects of peer support and mobile application-based walking programme on physical activity and physical function in rural older adults: a cluster randomized controlled trial. In: *EUROPEAN GERIATRIC MEDICINE* 13 (5), S. 1187–1195. DOI: 10.1007/s41999-022-00682-w.
4. Forster, Anne; Airlie, Jennifer; Birch, Karen; Cicero, Robert; Cundill, Bonnie; Ellwood, Alison et al. (2017): Research Exploring Physical Activity in Care Homes (REACH): study protocol for a randomised controlled trial. In: *TRIALS* 18 (1), S. 182. DOI: 10.1186/s13063-017-1921-8.
5. Harris, T.; Kerry, S.; Limb, E.; Furness, C.; Wahlich, C.; Victor, C. et al. (2018): Physical activity levels in adults and older adults 3-4 years after pedometer-based walking interventions:- long-term follow-up of participants from two randomised controlled trials in UK primary care. In: *JOURNAL OF PHYSICAL ACTIVITY & HEALTH* 15 (10), S82‐S83.
6. Harris, T.; Kerry, S.; Victor, C.; Ekelund, U.; Woodcock, A.; Iliffe, S. et al. (2013): Randomised controlled trial of a complex intervention by primary care nurses to increase walking in patients aged 60-74 years: protocol of the PACE-Lift (Pedometer Accelerometer Consultation Evaluation - Lift) trial. In: *BMC PUBLIC HEALTH* 13, S. 5. DOI: 10.1186/1471-2458-13-5.
7. Harris, T.; Kerry, S.; Victor, C.; Iliffe, S.; Ussher, M.; Fox-Rushby, J. et al. (2018): A pedometer-based walking intervention in 45- to 75-year-olds, with and without practice nurse support: the PACE-UP three-arm cluster RCT. In: *Health technology assessment (Winchester, England)* 22 (37), S. 1–274. DOI: 10.3310/hta22370.
8. Harris, T.; Kerry, S. M.; Limb, E. S.; Furness, C.; Wahlich, C.; Victor, C. R. et al. (2018): Physical activity levels in adults and older adults 3–4 years after pedometer-based walking interventions: long-term follow-up of participants from two randomised controlled trials in UK primary care. In: *PLOS MEDICINE* 15 (3). DOI: 10.1371/journal.pmed.1002526.
9. Harris, T.; Kerry, S. M.; Limb, E. S.; Victor, C. R.; Iliffe, S.; Ussher, M. et al. (2017): Effect of a Primary Care Walking Intervention with and without Nurse Support on Physical Activity Levels in 45- to 75-Year-Olds: the Pedometer And Consultation Evaluation (PACE-UP) Cluster Randomised Clinical Trial. In: *PLOS MEDICINE* 14 (1), e1002210. DOI: 10.1371/journal.pmed.1002210.
10. Harris, T.; Kerry, S. M.; Victor, C. R.; Ekelund, U.; Woodcock, A.; Iliff, S. et al. (2014): Does a complex intervention by primary care nurses increase walking in older people? Outcomes at 3 and 12 months in the PACE-Lift (Pedometer Accelerometer Consultation Evaluation-Lift) cluster-randomised controlled trial. In: *LANCET* 384, S. 3.
11. Harris, T.; Kerry, S. M.; Victor, C. R.; Ekelund, U.; Woodcock, A.; Iliffe, S. et al. (2015): A primary care nurse-delivered walking intervention in older adults: PACE (pedometer accelerometer consultation evaluation)-Lift cluster randomised controlled trial. In: *PLOS MEDICINE* 12 (2), e1001783. DOI: 10.1371/journal.pmed.1001783.
12. Harris PACE-UP (Pedometer and consultation evaluation--UP)--a pedometer-based walking intervention with and without practice nurse support in primary care patients aged 45-75 years: study protocol for a randomised controlled trial Trials 2013
13. Harris Effect of pedometer-based walking interventions on long-term health outcomes: prospective 4-year follow-up of two randomised controlled trials using routine primary care data PLoS Med 2019
14. Kerr, J.; Rosenberg, D.; Millstein, R. A.; Bolling, K.; Crist, K.; Takemoto, M. et al. (2018): Cluster randomized controlled trial of a multilevel physical activity intervention for older adults. In: *INTERNATIONAL JOURNAL OF BEHAVIORAL NUTRITION AND PHYSICAL ACTIVITY* 15 (1), S. 32. DOI: 10.1186/s12966-018-0658-4.
15. Kerr, Jacqueline; Rosenberg, Dori E.; Nathan, Andrea; Millstein, Rachel A.; Carlson, Jordan A.; Crist, Katie et al. (2012): Applying the ecological model of behavior change to a physical activity trial in retirement communities: description of the study protocol. In: *CONTEMPORARY CLINICAL TRIALS* 33 (6), S. 1180–1188. DOI: 10.1016/j.cct.2012.08.005.
16. Kim Text messaging to motivate walking in older African Americans: a randomized controlled trial Am J Prev Med 2013
17. LAUBACH, LLOYD; PORTER, KIMBER; HOVEY, PETER; LINDERMAN, J. O. N. (2009): A Modest Increase in Weekly Step Counts Improved Cardiovascular Function in Healthy Elderly Women. In: *Journal of Exercise Physiology Online* 12 (6), S. 25–32.
18. Lewis, Zakkoyya H.; Ottenbacher, Kenneth J.; Fisher, Steve R.; Jennings, Kristofer; Brown, Arleen F.; Swartz, Maria C.; Lyons, Elizabeth J. (2016): Testing Activity Monitors’ Effect on Health: Study Protocol for a Randomized Controlled Trial Among Older Primary Care Patients. In: *JMIR RESEARCH PROTOCOLS* 5 (2), e59. DOI: 10.2196/resprot.5454.
19. Limb, E.; Harris, T.; Kerry, S.; Victor, C.; Iliffe, S.; Ussher, M. et al. (2017): LONG-TERM OBJECTIVE PHYSICAL ACTIVITY DATA FROM TWO PRIMARY CARE PEDOMETER-BASED RANDOMISED CONTROLLED TRIALS IN MIDDLE-AGED AND OLDER ADULTS-ARE THERE STILL POSITIVE TRIAL EFFECTS AT 3 AND 4 YEARS? In: *JOURNAL OF EPIDEMIOLOGY AND COMMUNITY HEALTH* 71, A40‐A41. DOI: 10.1136/jech-2017-SSMAbstracts.80.
20. Liu Enhancing the Physical Activity Levels of Frail Older Adults with a Wearable Activity Tracker-Based Exercise Intervention: A Pilot Cluster Randomized Controlled Trial Int J Environ Res Public Health 2021
21. MacMillan, Freya; Fitzsimons, Claire; Black, Karen; Granat, Malcolm H.; Grant, Margaret P.; Grealy, Madeleine et al. (2011): West End Walkers 65+: a randomised controlled trial of a primary care-based walking intervention for older adults: study rationale and design. In: *BMC PUBLIC HEALTH* 11, S. 120. DOI: 10.1186/1471-2458-11-120.
22. Matz-Costa, Christina; Lubben, James; Lachman, Margie E.; Lee, Haenim; Choi, Yeon Jin (2018): A Pilot Randomized Trial of an Intervention to Enhance the Health-Promoting Effects of Older Adults’ Activity Portfolios: The Engaged4Life Program. In: *Journal of gerontological social work* 61 (8), S. 792–816. DOI: 10.1080/01634372.2018.1542371.
23. McMahon, S. K.; Lewis, B.; Oakes, J. M.; Wyman, J. F.; Guan, W. H.; Rothman, A. J. (2017): Assessing the Effects of Interpersonal and Intrapersonal Behavior Change Strategies on Physical Activity in Older Adults: a Factorial Experiment. In: *ANNALS OF BEHAVIORAL MEDICINE* 51 (3), S. 376–390. DOI: 10.1007/s12160-016-9863-z.
24. McMahon, Siobhan K.; Lewis, Beth A.; Guan, Weihua; Wyman, Jean F.; Rothman, Alexander J. (2021): Community-based intervention effects on older adults’ physical activity and falls: Protocol and rationale for a randomized optimization trial (Ready Steady3.0). In: *CONTEMPORARY CLINICAL TRIALS* 101, S. 106238. DOI: 10.1016/j.cct.2020.106238.
25. Mutrie, Nanette; Doolin, Orla; Fitzsimons, Claire F.; Grant, P. Margaret; Granat, Malcolm; Grealy, Madeleine et al. (2012): Increasing older adults’ walking through primary care: results of a pilot randomized controlled trial. In: *Family practice* 29 (6), S. 633–642. DOI: 10.1093/fampra/cms038.
26. NCT00183014 (2005): Caminemos! Trial to Increase Walking Among Sedentary Older Latinos. In: *https://clinicaltrials.gov/show/NCT00183014*.
27. NCT02146001 (2014): Reducing Sedentary Behavior vs. Increasing Physical Activity in Older Adults. In: *https://clinicaltrials.gov/show/NCT02146001*.
28. NCT03124537 (2017): Increasing Physical Activity Among Sedentary Older Adults: what, Where, When, and With Whom. In: *https://clinicaltrials.gov/show/NCT03124537*.
29. NCT03803085 (2019): Walking Competition to Enhance Daily Physical Activity and Social Engagement Among Older Adults. In: *https://clinicaltrials.gov/show/NCT03803085*.
30. NCT03906162 (2019): A 12-week Intervention With Motivational Interviewing and Physical Activity Monitoring, to Enhance the Daily Amount of Physical Activity in Community Dwelling Older Adults - a Randomized Controlled Trial. In: *https://clinicaltrials.gov/show/NCT03906162*.
31. NCT04235647 (2020): A Nurse Led Intervention to Promote Physical Activity. In: *https://clinicaltrials.gov/show/NCT04235647*.
32. Okamoto, N.; Nakatani, T.; Okamoto, Y.; Iwamoto, J.; Saeki, K.; Kurumatani, N. (2010): Increasing the Number of Steps Walked Each Day Improves Physical Fitness in Japanese Community-Dwelling Adults. In: *INTERNATIONAL JOURNAL OF SPORTS MEDICINE* 31 (4), S. 277–282. DOI: 10.1055/s-0029-1234057.
33. Paasche-Orlow, M.; Silliman, R.; Winter, M.; Cheng, D.; Henault, L.; Bickmore, T. (2012): Efficacy of a computer-based intervention to promote walking in older adults. In: *JOURNAL OF THE AMERICAN GERIATRICS SOCIETY* 60, S4‐. DOI: 10.1111/j.1532-5415.2012.04000.x.
34. Pelssers, Johan; Delecluse, Christophe; Opdenacker, Joke; Kennis, Eva; van Roie, Evelien; Boen, Filip (2013): Every step counts!": effects of a structured walking intervention in a community-based senior organization. In: *JOURNAL OF AGING AND PHYSICAL ACTIVITY* 21 (2), S. 167–185. DOI: 10.1123/japa.21.2.167.
35. RBR-62sggb (2020): Educational program to promote physical activity, cognitive and cardiorespiratory performance in sedentary older adults. In: *https://trialsearch.who.int/Trial2.aspx?TrialID=RBR-62sggb*.
36. Recio-Rodríguez, José I.; Gonzalez-Sanchez, Susana; Tamayo-Morales, Olaya; Gómez-Marcos, Manuel A.; Garcia-Ortiz, Luis; Niño-Martín, Virtudes et al. (2022): Changes in lifestyles, cognitive impairment, quality of life and activity day living after combined use of smartphone and smartband technology: a randomized clinical trial (EVIDENT-Age study). In: *BMC GERIATRICS* 22 (1), S. 782. DOI: 10.1186/s12877-022-03487-5.
37. Recio-Rodríguez, José I.; Lugones-Sanchez, Cristina; Agudo-Conde, Cristina; González-Sánchez, Jesús; Tamayo-Morales, Olaya; Gonzalez-Sanchez, Susana et al. (2019): Combined use of smartphone and smartband technology in the improvement of lifestyles in the adult population over 65 years: study protocol for a randomized clinical trial (EVIDENT-Age study). In: *BMC GERIATRICS* 19 (1), S. 19. DOI: 10.1186/s12877-019-1037-y.
38. Schmidt, Laura I.; Jansen, Carl-Philipp; Depenbusch, Johanna; Gabrian, Martina; Sieverding, Monika; Wahl, Hans-Werner (2022): Using wearables to promote physical activity in old age : Feasibility, benefits, and user friendliness. In: *Zeitschrift fur Gerontologie und Geriatrie* 55 (5), S. 388–393. DOI: 10.1007/s00391-022-02083-x.
39. Takahashi, Masaki; Lim, Pei Jean; Tsubosaka, Miku; Kim, Hyeon-Ki; Miyashita, Masashi; Suzuki, Katsuhiko et al. (2019): Effects of increased daily physical activity on mental health and depression biomarkers in postmenopausal women. In: *JOURNAL OF PHYSICAL THERAPY SCIENCE* 31 (4), S. 408–413. DOI: 10.1589/jpts.31.408.
40. Thompson. "Go4Life" exercise counseling, accelerometer feedback, and activity levels in older people. Arch Gerontol Geriatr. 2014 May-Jun;58(3):314-9. doi: 10.1016/j.archger.2014.01.004.
41. van Hoecke, Ann-Sophie; Delecluse, Christophe; an Bogaerts; Boen, Filip (2014): The Long-Term Effectiveness of Need-Supportive Physical Activity Counseling Compared With a Standard Referral in Sedentary Older Adults. In: *Journal of Aging & Physical Activity* 22 (2), S. 186–198.
42. Wijsman. Effects of a web-based intervention on physical activity and metabolism in older adults: randomized controlled trial. J Med Internet Res. 2013 Nov 6;15(11):e233. doi: 10.2196/jmir.2843.
