# Supplementary material for: Efficacy of activity tracker-based interventions and their behavioral components in promoting physical activity and reducing sedentary behavior in older adults: a systematic review of randomized controlled trials
Source: Eur Rev Aging Phys Act. 2026 Jan 12;23:5. doi: 10.1186/s11556-025-00396-5 (PMC12853638; doi:10.1186/s11556-025-00396-5)
Supplement: Supplementary file 6 — Additional file 6. References of excluded studies – Outcome. [file 11556_2025_396_MOESM6_ESM.docx]

# Additional File 6. References of excluded studies – Outcome

# Reason for exclusion: Outcome

1. Auerswald, Tina; Hendker, Anna; Ratz, Tiara; Lippke, Sonia; Pischke, Claudia R.; Peters, Manuela et al. (2022): Impact of Activity Tracker Usage in Combination with a Physical Activity Intervention on Physical and Cognitive Parameters in Healthy Adults Aged 60+: A Randomized Controlled Trial. In: *INTERNATIONAL JOURNAL OF ENVIRONMENTAL RESEARCH AND PUBLIC HEALTH* 19 (7). DOI: 10.3390/ijerph19073785.
2. Bao, Tian; Carender, Wendy J.; Kinnaird, Catherine; Barone, Vincent J.; Peethambaran, Geeta; Whitney, Susan L. et al. (2018): Effects of long-term balance training with vibrotactile sensory augmentation among community-dwelling healthy older adults: a randomized preliminary study. In: *JOURNAL OF NEUROENGINEERING AND REHABILITATION* 15 (1), S. 5. DOI: 10.1186/s12984-017-0339-6.
3. Haeger, Mathias; Bock, Otmar; Zijlstra, Wiebren (2021): Smartphone-based health promotion in old age : An explorative multi-component approach to improving health in old age. In: *Zeitschrift fur Gerontologie und Geriatrie* 54 (2), S. 146–151. DOI: 10.1007/s00391-020-01700-x.
4. ISRCTN17158017 (2021): Improving health and maintaining independent living in frail older adults by reducing sitting time. In: *https://trialsearch.who.int/Trial2.aspx?TrialID=ISRCTN17158017*.
5. Kariuki, Jacob K.; Gibbs, Bethany B.; Erickson, Kirk I.; Kriska, Andrea; Sereika, Susan; Ogutu, David et al. (2021): The feasibility and acceptability of a web-based physical activity for the heart (PATH) intervention designed to reduce the risk of heart disease among inactive African Americans: Protocol for a pilot randomized controlled trial. In: *CONTEMPORARY CLINICAL TRIALS* 104, S. 106380. DOI: 10.1016/j.cct.2021.106380.
6. Nebeker, C.; Zlatar, Z. Z. (2021): Learning From Older Adults to Promote Independent Physical Activity Using Mobile Health (mHealth). In: *FRONTIERS IN PUBLIC HEALTH* 9. DOI: 10.3389/fpubh.2021.703910.
7. Piau, Antoine; Steinmeyer, Zara; Charlon, Yoann; Courbet, Laetitia; Rialle, Vincent; Lepage, Benoit et al. (2021): A Smart Shoe Insole to Monitor Frail Older Adults’ Walking Speed: Results of Two Evaluation Phases Completed in a Living Lab and Through a 12-Week Pilot Study. In: *JMIR MHEALTH AND UHEALTH* 9 (7), e15641. DOI: 10.2196/15641.
8. Richeson, Nancy E.; Croteau, Karen A.; Jones, David B.; Farmer, Bonnie Cashin (2006): Effects of a Pedometer-Based Intervention on the Physical Performance and Mobility-Related Self-Efficacy of Community-Dwelling Older Adults: An Interdisciplinary Preventive Health Care Intervention. In: *Therapeutic Recreation Journal* 40 (1), S. 18–32.
9. Sjögren, P.; Fisher, R.; Kallings, L.; Svenson, U.; Roos, G.; Hellénius, M. L. (2014): Stand up for health‐avoiding sedentary behaviour might lengthen your telomeres: secondary outcomes from a physical activity RCT in older people. In: *BRITISH JOURNAL OF SPORTS MEDICINE* 48 (19), S. 1407–1409. DOI: 10.1136/bjsports-2013-093342.
