# Supplementary material for: Efficacy of activity tracker-based interventions and their behavioral components in promoting physical activity and reducing sedentary behavior in older adults: a systematic review of randomized controlled trials
Source: Eur Rev Aging Phys Act. 2026 Jan 12;23:5. doi: 10.1186/s11556-025-00396-5 (PMC12853638; doi:10.1186/s11556-025-00396-5)
Supplement: Supplementary file 7 — Additional file 7. References of excluded studies – Study design. [file 11556_2025_396_MOESM7_ESM.docx]

# Additional file 7. References of excluded studies – Study design

# Reason for exclusion: Study design

1. Nishiwaki, Masato; Fujibayashi, Mami; Nanayama, Chika; Ogawa, Noriko; Itakura, Isako; Matsumoto, Naoyuki (2018): Increasing levels of daily physical activity for arterial stiffness reduction in older women: a community-based pilot study. In: *The Journal of sports medicine and physical fitness* 58 (11), S. 1701–1709. DOI: 10.23736/s0022-4707.17.07238-3.
2. Nyman, Samuel R.; Goodwin, Kelly; Kwasnicka, Dominika; Callaway, Andrew (2016): Increasing walking among older people: A test of behaviour change techniques using factorial randomised N-of-1 trials. In: *PSYCHOLOGY & HEALTH* 31 (3), S. 313–330. DOI: 10.1080/08870446.2015.1088014.
3. Tiedemann, Anne; Hassett, Leanne; Sherrington, Catherine (2015): A novel approach to the issue of physical inactivity in older age. In: *PREVENTIVE MEDICINE REPORTS* 2, S. 595–597. DOI: 10.1016/j.pmedr.2015.07.008.
4. JMA-IIA00062 (2011): Pedometer-based walking program to improve physical and cognitive function in community-dwelling older adults. In: *https://trialsearch.who.int/Trial2.aspx?TrialID=ISRCTN37185489*.
