# Supplementary material for: Efficacy of activity tracker-based interventions and their behavioral components in promoting physical activity and reducing sedentary behavior in older adults: a systematic review of randomized controlled trials
Source: Eur Rev Aging Phys Act. 2026 Jan 12;23:5. doi: 10.1186/s11556-025-00396-5 (PMC12853638; doi:10.1186/s11556-025-00396-5)
Supplement: Supplementary file 8 — Additional file 8. Characteristics of ongoing studies. [file 11556_2025_396_MOESM8_ESM.docx]

**Additional file 8.** Characteristics of ongoing studies

**No 1: ACTRN12617001186347 (trial registry entry)**

| **Methods** | Study design | Randomized controlled trial |
| --- | --- | --- |
| **Participants** | Main inclusion criteria: | - Screen falls risk positive (scores 4 or higher on the STEADI falls risk self-assessment tool) - Rapid cognitive screen of 6 - Able to walk independently up to 10m with or without walking aid - Able to converse in English - Score of 1 or more on FRAIL screen |
|  | Setting: | Community |
|  | Age: | 65+ years |
|  | Country/ies: | Australia |
| **Interventions** | Intervention(s): | - Accelerometer Dose: 1 week - Pedometer Dose: 12 weeks - ‘Choose Health: Be Active’ booklet |
|  | Comparator(s): | Written information about the benefits of being active (i.e. the ‘Choose Health: Be Active’ booklet) |
|  | Duration of follow-up: | 6 months |
| **Outcome related to PA AND/OR SB** | Outcome 1 | Total sedentary time |
|  | How measured: | Accelerometer |
|  | Time points measured: | Baseline, week 12, week 24 |
|  | Time points reported: | NR |
| **Starting date** | Trial start date: | 14/08/2017 |
|  | Trial completion date: | NR |
| **Contact information** |  | Dr Kareeann Khow  Adelaide G-TRAC Centre  61 Silkes Road, Paradise SA 5075  Australia  Phone: +61883132144 Email: kareeann.khow@adelaide.edu.au |
| **Notes** | Trial identifier: (e.g. NCT...) | ACTRN12617001186347 |
|  | Funding: | Adelaide Geriatric Research and Training with Aged Care (G-TRAC) with University of Adelaide |
|  | Conflict of interests: | NR |

**No 2: Khow et al., 2018 (protocol paper)**

| **Methods** | Study design | Randomized controlled trial |
| --- | --- | --- |
| **Participants** | Main inclusion criteria: | - Screen falls risk positive (scores 4 or higher on the STEADI falls risk self-assessment tool) - Rapid cognitive screen of 6 - Able to walk independently up to 10m with or without walking aid - Able to converse in English - Score of 1 or more on FRAIL screen |
|  | Setting: | Community |
|  | Age: | 65+ years |
|  | Country/ies: | Australia |
| **Interventions** | Intervention(s): | - Provision of objective information regarding their sedentary levels based on accelerometer recordings - Education - Two face-to-face health coaching sessions - Pedometer   dose: weeks 0 and 6 |
|  | Comparator(s): | - Written information about the benefits of being active (i.e. the ‘Choose Health: Be Active’ booklet) - Provision of their baseline status from the first accelerometer assessment |
|  | Duration of follow-up: | 6 months |
| **Outcome related to PA AND/OR SB** | Outcome 1 | Sedentary time |
|  | How measured: | ActivPal |
|  | Time points measured: | Baseline, week 12, week 24 |
|  | Time points reported: | NR |
| **Starting date** | Trial start date: | 14/08/2017 |
|  | Trial completion date: | NR |
| **Contact information** |  | Dr Kareeann Khow  Adelaide G-TRAC Centre  61 Silkes Road, Paradise SA 5075  Australia  Phone: +61883132144  Email: kareeann.khow@adelaide.edu.au |
| **Notes** | Trial identifier: (e.g. NCT...) | ACTRN12617001186347 |
|  | Funding: | Adelaide Geriatric Research and Training with Aged Care (G-TRAC) with University of Adelaide |
|  | Conflict of interests: | NR |

**No 3:** **NCT03417440 (trial registry entry)**

| **Methods** | Study design | Factorial randomized controlled trial |
| --- | --- | --- |
| **Participants** | Main inclusion criteria: | - score ≥5 on a 6-item cognitive screener - report <150 minutes of moderate-to-vigorous PA/week as per a single-item screener - ambulatory - ability to safely participate in PA as determined by self-report of ability to regularly walk safely - smartphone owner for ≥1 month + observed ability to reliably access and operate a smartphone - available by telephone for the duration of either study - English speaking |
|  | Setting: | Community |
|  | Age: | 65-84 years |
|  | Country/ies: | Los Angeles or Pacific Time Zone |
| **Interventions** | Intervention(s): | PA-tracking app  Dose: 2 weeks + 4 months |
|  | Comparator(s): | Active |
|  | Duration of follow-up: | 4 months |
| **Outcome related to PA AND/OR SB** | Outcome 1 | Objective PA level |
|  | How measured: | activePal |
|  | Time points measured: | 3-day monitoring period pre and post intervention |
|  | Time points reported: | NR |
|  | Outcome 2 | Self-reported PA level |
|  | How measured: | Physical Activity Scale |
|  | Time points measured: | pre and post intervention |
|  | Time points reported: | NR |
|  | Outcome 3 | Objective Sedentary Time |
|  | How measured: | activePal |
|  | Time points measured: | 3-day monitoring period pre and post intervention |
|  | Time points reported: | NR |
| **Starting date** | Trial start date: | 10/09/2020 |
|  | Trial completion date: | NR |
| **Contact information** |  | Stacey L. Schepens Niemiec, PhD  USC Chan Division of Occupational Science and Occupational Therapy  1540 Alcazar St., CHP-133  Los Angeles, CA |
| **Notes** | Trial identifier: (e.g. NCT...) | NCT03417440 |
|  | Funding: | NIH / National Institute on Aging (NIA)  NIH / NIA (Alzheimer’s Disease and Related Dementias Administrative Supplement) |
|  | Conflict of interests: | NR |
