# Supplementary material for: Efficacy of activity tracker-based interventions and their behavioral components in promoting physical activity and reducing sedentary behavior in older adults: a systematic review of randomized controlled trials
Source: Eur Rev Aging Phys Act. 2026 Jan 12;23:5. doi: 10.1186/s11556-025-00396-5 (PMC12853638; doi:10.1186/s11556-025-00396-5)
Supplement: Supplementary file 9 — Additional file 9. Characteristics of studies awaiting classification. [file 11556_2025_396_MOESM9_ESM.docx]

**Additional file 9.** Characteristics of studies awaiting classification

Reasons for the studies being classified as ‘awaiting classification’ in *Italics*

**No 1: NCT01158638 (trial registry entry)**

| **Methods** | Study design | Randomized controlled trial |
| --- | --- | --- |
| **Participants** | Main inclusion criteria: | - Sufficient physical function (Habitual gait speed ≥ 1.5 mph, AND No use of a cane or walker, AND Able to walk ≥ 50 feet unaided) - Sufficient cognitive function (mini-mental state examination >23 score) - Be able to read and write in English - Sedentary behavior (no regular activity/ exercise for previous 6 month) |
|  | Setting: | Community |
|  | Age: | *50-85 years* |
|  | Country/ies: | United States |
| **Interventions** | Intervention(s): | Behavioral: Web Mediated Step Intervention  Dose: NR |
|  | Comparator(s): | Usual Care |
|  | Duration of follow-up: | NR |
| **Outcomes related to PA AND/OR SB** | Outcome 1 | Objectively assessed steps per day |
|  | How measured: | Pedometer determined physical activity |
|  | Time points measured: |  |
|  | Time points reported: | 12 weeks |
| **Starting date** | Trial start date: | 07/07/2010 |
|  | Trial completion date: | NR |
| **Contact information** |  | Scott J. Strath Physical Activity & Health Research Laboratory  University of Wisconsin, Milwaukee |
| **Notes** | Trial identifier: | NCT01158638 |
|  | Funding: | NR |
|  | Conflict of interests: | NR |
