# Supplementary material for: Efficacy of activity tracker-based interventions and their behavioral components in promoting physical activity and reducing sedentary behavior in older adults: a systematic review of randomized controlled trials
Source: Eur Rev Aging Phys Act. 2026 Jan 12;23:5. doi: 10.1186/s11556-025-00396-5 (PMC12853638; doi:10.1186/s11556-025-00396-5)
Supplement: Supplementary file 10 — Additional file 10. Overview of Behavior Change Techniques (BCTs) applied in activity tracker interventions. [file 11556_2025_396_MOESM10_ESM.docx]

**Additional file 10.** Behavior Change Techniques (BCTs) implemented in activity tracker interventions operationalized according to the BCT Taxonomy (v1) by Michie et al., 2013

| **Author (year)** | **Group of BCTs** | **BCT Code** | **Operationalization of BCTs (description of how each technique was implemented within the activity tracker intervention)** |
| --- | --- | --- | --- |
| Alley et al. (2022) | 1. Goals and planning  2. Feedback and monitoring  3. Social support  7. Associations  10. Reward and threat | 1.2. Problem solving  1.3. Goal setting (outcome)  1.4. Action planning  2.2. Feedback on behavior  2.3. Self-monitoring of behavior  2.7. Feedback on outcome(s) of behavior  3.1. Social support (unspecified)  7.1. Prompts/cues  10.3. Non-specific reward | Problem solving   - tailored advice to relapse prevention   Goal setting   - meeting the PA recommendation, participants were also encouraged to limit their sitting time to <8 hours per day and to take regular breaks from sitting   Action planning   - intervention website includes an action planning tool that participants are encouraged to complete - tool guides participants in setting an action plan (what, where, when, and with whom) for being active in the following fortnight   Feedback on behavior   - tailored advice was computer automated and used activity tracker data to select appropriate messages from a database of messages using if-then algorithms (e.g., if low self-efficacy and inactive then message on improving self-efficacy by starting small) - PA advice was tailored to participants’ characteristics and environment, PA behavior, and psychosocial correlates of PA - LED lights individually lit up for every 2000 steps taken in a day   Self-monitoring   - the activity tracker did not have a display except for five LED lights which individually light up for every 2000 steps taken in a day - monitoring of individual PA data with a web-based application   Social support   - was stated as an intervention component without further information   Prompts/cues   - were stated as an intervention component without further information   Rewards   - were stated as an intervention component without further information |
| Bouchard et al. (2013) | 1. Goals and planning  2. Feedback and monitoring  3. Social support  4. Shaping knowledge  8. Repetition and substitution | 1.3. Goal setting (outcome)  1.4. Action planning  1.7. Review outcome goal(s)  2.2. feedback on behavior  2.4. Self-monitoring of outcome(s) of behavior  2.7. Feedback on outcome(s) of behavior  3.2. Social support (practical)  4.1. Instruction on how to perform the behavior  8.1. Behavioral practice/rehearsal | Goal setting   - participants were instructed to reach the minimal intensity every time they were doing PA for a minimum of 10 minutes - in terms of duration, their objective was to increase it to a minimum of 75 minutes per week after 4 weeks and a minimum of 150 minutes after 8 weeks   Action planning   - strategies to reach these goals were specified when each participant detailed a specific, measurable, attainable, realistic, and time-sensitive (SMART) goal for the next eight weeks   Review outcome goal(s)   - goals were revised in a follow-up phone conversation 4 weeks after the information/training session   Self-monitoring   - participants were instructed to pay attention to the self-perceived exertion to identify the correct intensity when not wearing the device at the end of the intervention   Feedback on behavior   - participants were contacted by phone after 4 weeks to increase compliance and answer questions regarding the provided strategy   Social support   - those who were interested could exchange their contact information to walk together   Behavioral practice/rehearsal   - every group was given 30 minutes to walk on a flat surface to test their tool and ask questions of the staff - pedometer group was instructed to target 100 steps per minute to reach at least moderate intensity |
| Brickwood et al. (2021) | 1. Goals and planning  2. Feedback and monitoring | - 1. Goal setting (outcome)   1.7. Review outcome goal(s)  2.2 Feedback on behavior  2.3 Self-monitoring of behavior  2.4. Self-monitoring of outcome(s) of behavior  2.7. Feedback on outcome(s) of behavior | Goal setting   - daily step goal was individually prescribed for each participant based on their physical function and current level of PA - daily step goal was slightly adjusted (±200-500 steps) during the weekly text message feedback from an accredited exercise physiologist based on the previous week’s step data   Review outcome goal(s)   - if participants continued to significantly underachieve or exceed their initial daily step goal, the app was adjusted during their 3-, 6-, or 12-month assessment   Feedback on behavior   - automated feedback provided by the tracker and app based on daily step goal - in addition to daily feedback available through the app, participants received weekly, personalized text messages from an accredited exercise physiologist - the text message contained feedback related to average daily steps and a comparison of their daily step goal with that of the previous week   Self-monitoring   - participants were asked to synchronize the tracker with the app at the end of each day but could check the progress toward their daily step goal as desired |
| Croteau et al. (2004) | 1. Goals and planning  2. Feedback and monitoring | 1.3. Goal setting (outcome)  1.4. Action planning  1.7. Review outcome goal(s)  2.2. Feedback on behavior  2.7. Feedback on outcome(s) of behavior | Goal setting   - consisted of examining baseline goals and determining the rate of steps/day increase desired - goals were individualized, with participants aiming for a 5% increase in daily steps each week if they met their previous goals   Action planning   - participants used self-selected strategies to increase daily PA - strategies used to increase PA in the previous week were recorded and the same and/or new strategies were selected for the next week   Review outcome goal(s)   - during weekly meetings, goals and strategies developed during the initial counseling session were discussed and revised, if necessary   Feedback on behavior   - if a participant met his or her goal for the previous week, a new goal was set for the following week, if a participant did not meet his/her goal for the previous week the participant was instructed to keep the same goal for the following week |
| Croteau et al. (2007) | 1. Goals and planning  2. Feedback and monitoring  7. Associations | 1.3. Goal setting (outcome)  1.4. Action planning  1.7. Review outcome goal(s)  2.4. Self-monitoring of outcome(s) of behavior  2.2. Feedback on behavior  2.7. Feedback on outcome(s) of behavior  7.1. Prompts/cues | Goal setting   - participants met individually with a facilitator to set daily step goals - initial daily step goals were set as a percentage of the participant’s baseline step counts and involved an increase of 5% over baseline   Action planning   - participants met individually with a facilitator to select strategies for increasing daily step counts - from a list of sample strategies and through brainstorming, participants selected strategies they wanted to use to increase their daily PA   Review outcome goal(s)   - prior to starting the 12-week maintenance phase, participants met individually with a facilitator to review the process for setting daily step goals, procedures for keeping the step calendar, and strategies to increase and/or maintain PA   Feedback on behavior   - examining the step counts on the pedometer provided feedback on the amount of PA attained   Self-monitoring   - intervention group participants met individually with a facilitator to discuss procedures for keeping a step calendar - at the end of each day participants record the following on their step calendar: date, total number of steps displayed on the pedometer, and strategies used to increase PA that day   Prompts/cues   - wearing the pedometer served as a cue or reminder to increase walking activity |
| Koizumi et al. (2009) | 1. Goals and planning  2. Feedback and monitoring | 1.3. Goal setting (outcome)  2.2. Feedback on behavior  2.3. Self-monitoring of behavior  2.7. Feedback on outcome(s) of behavior | Goal setting   - participants were recommended to accumulate 9000 steps and 30 minutes of moderate intensity PA per day   Feedback on behavior   - feedback based on accelerometer daily PA data (number of daily steps, time spent performing daily moderate intensity physical activity) was provided to each participant every two weeks - based on activity level, recommendations were provided to each participant (e.g., increase STEPS by 500 and engage in 3 more minutes of MPA per day)   Self-monitoring   - participants were provided with graphs and scatter plots representing their activity levels for the previous two weeks |
| Kolt et al. (2012) | 1. Goals and planning  2. Feedback and monitoring  3. Social support | 1.1. Goal setting (behavior)  1.2. Problem solving  1.3. Goal setting (outcome)  1.4. Action planning  2.2. Feedback on behavior  2.4. Self-monitoring of outcome(s) of behavior  2.7. Feedback on outcome(s) of behavior  3.3. Social support (emotional) | Goal setting   - goal setting based on steps was used as a main component of this intervention - individual goals were pre-dominantly based on pursuing activities that increase step counts in an incremental manner over time, and were dependent on identified lifestyle factors, accessibility to facilities, level of mobility, and current level of activity - some goals were specifically based on engaging in an activity and receiving feedback via the pedometer on step-based gains, whereas other goals were based on increasing the number of steps per day   Problem solving   - telephone counselling used principals of motivational interviewing such as assisting patients to identify barriers and solutions to physical activity participation, and discussing positive aspects of participation in activity and negative aspects of not participating in enough activity   Action planning   - goals were set by participants as part of their interaction with the PA counselor, in which they received guidance on how to set relevant goals based on identified barriers and on factors that enable increasing activity   Feedback on behavior   - the pedometer was used to provide immediate feedback to the participants regarding their accumulation of activity towards daily goal   Self-monitoring   - participants were encouraged to use their pedometer to monitor steps taken throughout the day   Social support   - telephone counselling used principals of motivational interviewing such as assisting patients to identify barriers and solutions to physical activity participation, and discussing positive aspects of participation in activity and negative aspects of not participating in enough activity |
| Kwan et al. (2020) | 1. Goals and planning  2. Feedback and monitoring  10. Reward and threat | 1.3. Goal setting (outcome)  2.2. Feedback on behavior  2.3. Self-monitoring of behavior  2.4. Self-monitoring of outcome(s) of behavior  2.7. Feedback on outcome(s) of behavior  10.4. Social reward | Goal setting   - personalized PA goals were set based on practice availability, baseline fitness, previous performance, and personal wishes - weekly training goals were tailored according to the participants’ individual level of physical fitness at baseline and to their progress (e.g., relating to walking speed, walking time, number of steps)   Feedback on behavior, Self-monitoring   - provision of self-tracking of the walking behaviors and the amounts of PA with immediate feedback - activity tracker autonomously and continually monitored the walking behavior of the participants (e.g., steps, walking speed, walking time, PA intensity) - messages were sent to the participants at least once a week (e.g., praise messages, e-reminders, personalized goals, coaching)   Self-monitoring   - provision of self-tracking of the walking behaviors and the amounts of PA with immediate feedback - activity tracker autonomously and continually monitored the walking behavior of the participants (e.g., steps, walking speed, walking time, PA intensity)   Rewards   - messages were sent to the participants at least once a week (e.g., praise messages, e-reminders, personalized goals, coaching) - activity tracker provided immediate rewards (no further information specified) |
| Leskinen et al. (2021) | 1. Goals and planning  2. Feedback and monitoring  5. Natural consequences  7. Associations  10. Reward and threat | 1.1. Goal setting (behavior)  1.3. Goal setting (outcome)  2.2. Feedback on behavior  2.3. Self-monitoring of behavior  2.7. Feedback on outcome(s) of behavior  5.1. Information about health consequences  7.1. Prompts/cues  10.4. Social reward | Goal setting   - daily activity goal attainment was chosen as the behavioral target for the intervention in order to maintain concordance between the goals and the means of the intervention - participants were instructed to pursue the daily activity goal, initially set at stage 1 as per the goals set by the tracker manufacture, preset stages in activity goals were built around user’s typical daily activities, and they were also sensitive to the user’s gender and age - at stage 1, the amount of daily activity necessary to achieve the goal exceeded the recommendation of weekly 150 min of MVPA, various kinds of activities contribute to the achieving of the daily activity goal - participants who frequently achieved or exceeded 100% of their daily activity goals at stage 1 were suggested by the re-searcher, via e-mail or SMS, to move on to stage 2 (activity goal comparable with 3 hours of walking) and ultimately stage 3 (activity goal comparable with 3,5 hours of walking) - no further counseling or guidance on how to achieve the daily activity goal was given to the participants   Feedback on behavior   - based on the accumulated daily activity, the tracker provided feedback and displayed practical guidance on how to reach the remaining part of the daily goal - activity tracker provided feedback on the attainment of the daily activity goal, and if the tracker had been worn sufficiently, a detailed feedback on the health benefits of accumulated activity, sedentary time, and sleep on daily, weekly, and monthly levels   Self-monitoring   - activity tracker enabled the user to monitor the real-time achievement of the activity goal and, e.g., the accumulation of daily steps - activity tracker displayed overviews and summaries of the activity data on a daily, weekly, and monthly basis   Prompts/cues   - the tracker gave an inactivity alert by vibrating after 55 min of a nonmovement period, coupled with a prompt “it’s time to move” shown on the display   Information about health consequences   - provision of detailed feedback on the health benefits of accumulated activity, sedentary time, and sleep on daily, weekly, and monthly levels   Reward   - upon 100% fulfillment of the daily goal, the tracker congratulated the user |
| McLellan et al. (2018) | 1. Goals and planning  2. Feedback and monitoring  4. Shaping knowledge  8. Repetition and substitution  10. Reward and threat | 1.3. Goal setting (outcome)  2.2. Feedback on behavior  4.1. Instruction on how to perform the behavior  8.1. Behavioral practice/rehearsal  10.4. Social reward | Goal setting   - the goal was 150 minutes PA per week, reaching at least moderate intensity in bouts of 10 minutes or more - intensity was individually determined upon completion of a fitness test   Feedback on behavior   - pedometer indicated the walking cadence needed to reach PA intensity - after a participant completed one bout of at least 10 minutes PA at the minimum required intensity, a star was shown on the pedometer display   Instruction on how to perform the behavior   - participants attended a total of three walking visits in the first week to simply walk with a research assistant for 50 minutes around an indoor track |
| McMurdo et al. (2010) | 1. Goals and planning  2. Feedback and monitoring  3. Social support | 1.1. Goal setting (behavior)  1.4. Action planning  2.4. Self-monitoring of outcome(s) of behavior  3.3. Social support (emotional) | Goal setting   - a graded goal-setting approach to increase walking was given, with clear advice on when and where to walk and how to schedule time for PA   Action planning   - theory-based advice was given to each participant in the form of individualized activity action plans and plans to address barriers to action   Self-monitoring   - participants were given monthly daily activity diaries to complete with logs of pedometer step counts or minutes spent walking outdoors   Social support   - participants were contacted over the telephone once a week for the first month, then every 2 weeks for 2 months, and then monthly until the end of the 6-month study to provide motivation and encouragement and to trouble-shoot any problem |
| Muellmann  et al. (2019) | 1. Goals and planning  2. Feedback and monitoring  3. Social support  4. Shaping knowledge  5. Natural consequences  6. Comparison of behavior  10. Reward and threat | 1.2. Problem solving  1.3. Goal setting (outcome)  2.3. Self-monitoring of behavior  2.7. Feedback on outcome(s) of behavior  3.1. Social support (unspecified)  3.2. Social support (practical)  4.1. Instruction on how to perform the behavior  5.1. Information about health  Consequences  10.10. Reward (outcome) | Problem solving   - during weekly group meetings participants discussed different factors which may enhance PA promotion (e.g., social support, the use of action planning, and strategies for PA maintenance)   Goal setting   - according to the PA recommendations, participants were instructed to engage in exercises to improve balance (two times per week), flexibility (two times per week), strength (on two or more nonconsecutive days per week involving major muscle groups), and endurance (for at least 150 min with moderate-to-vigorous intensity per week in bouts of 10 min)   Self-monitoring   - access to a web-based PA diary to track PA behavior over the ten-week intervention period, activity tracker data were synchronized with the website   Feedback on behavior   - website provided weekly feedback on whether PA goals were reached   Social support   - participants received the opportunity to network with other intervention participants via an invite friend's function and an online forum   Instructions on how to perform the behavior   - weekly group meetings were offered in which participants practiced PA in groups (e.g., community walks)   Information about health consequences   - weekly group meetings were offered, in which participants received health education regarding the role of PA for healthy ageing   Reward   - participants received goal-specific rewards (digital cups)   Comparison of behavior   - stated as an intervention component without further information |
| Roberts et al. (2019) | 1. Goals and planning  2. Feedback and monitoring  3. Social support | 1.3. Goal setting (outcome)  1.4. Action planning  2.3. Self-monitoring of behavior  2.7. Feedback on outcome(s) of behavior  3.3. Social support (emotional) | Goal setting   - individualized goal for non-exercise PA minutes     Action planning   - a behavioral specialist assists with goal setting of non-exercise PA minutes and includes considerable attention placed on the process of how non-exercise PA might be increased given the daily demands and environmental constraints of the individual   Self-monitoring   - adherence to monitor wearing was tracked by participants via a wear log to record all times the device is not worn   Feedback on behavior   - study team monitored participants’ daily non-exercise PA and communicated weekly (at the exercise intervention visits or by phone, depending on phase of the study) to provide additional motivation and individual goal-based strategies for increasing non-exercise PA   Social support   - participants received cognitive-behavioral counseling that focused on reducing SB and increasing non-exercise PA in their daily life - behavioral counseling was individualized to each participant on how they could strategize an increase in non-exercise PA |
| Rowley et al. (2019) | 1. Goals and planning  2. Feedback and monitoring  3. Social support  5. Natural consequences  10. Reward and threat | 1.2. Problem solving  1.3. Goal setting (outcome)  1.4. Action planning  2.2. Feedback on behavior  2.4. Self-monitoring of outcome(s) of behavior  2.7 Feedback on outcome(s) of behavior  3.1. Social support (unspecified)  5.1. Information about health  Consequences  10.10. Reward (outcome) | Problem solving   - motivational messages were designed to offer strategies for overcoming user-identified barriers to attaining their PA step goal - if the participant was not in compliance, the user was guided through a series of interactive screens that were designed to collect barriers to accomplishing the goal and then deliver motivational messages tagged and retrieved from a database library   Goal setting   - goal to increase daily step count by 10% each week until they met 10,000 steps per day after which they were instructed to maintain 10,000 steps per day   Action planning   - interactive website employed key strategies to increase PA systematically   Feedback on behavior   - graphical representations of daily steps were provided along with information on how well they corresponded with intrinsically set goals   Self-monitoring   - uploaded steps per day were graphically represented for each day for the prior week, and plotted against nationally recommended amounts - daily step counts were recorded on paper logs and mailed back to researchers each week   Social support   - users were guided by an ongoing discussion forum, posing questions and solutions to increase PA, and access to “ask the expert” (a trained behaviorist and member of the research team)   Information about health consequences   - cognitive understanding of the benefits of PA, education on national recommendations, and self-awareness of current activity levels   Reward   - if a participant was in compliance, the user was guided through a series of congratulatory screens and a directive for setting the upcoming week’s PA step goal |
| Slaght et al. (2017) | 1. Goals and planning  2. Feedback and monitoring  4. Shaping knowledge  6. Comparison of behavior  8. Repetition and substitution | 1.3. Goal setting (outcome)  2.2. Feedback on behavior  2.4. Self-monitoring of outcome(s) of behavior  2.5. Monitoring of outcome(s) of behavior without feedback  4.1. Instruction on how to perform the behavior  6.1. Demonstration of the behavior  8.1. Behavioral practice/ rehearsal | Goal setting   - goal = walk 150 minutes per week, reaching at least moderate intensity in 10-minute bouts - intensity was individually determined upon completion of a cardiorespiratory fitness test   Feedback on behavior   - pedometer displayed a star every time the participant completed one 10-minute bout at the minimum required intensity (visual feedback)   Self-monitoring   - pedometer screen displayed total steps accumulated each day - all participants were asked to self-report the time they spent walking each day using a logbook - participants also recorded MVPA time each day and the total number of 10-min bouts accumulated from the pedometer   Instruction on how to perform the behavior   - participants were shown how to reach moderate intensity based on their prescribed walking cadence by checking the pedometer after 10 min of consecutive walking to ensure the intensity was being achieved |
| Sugden et al. (2008) | 1. Goals and planning  2. Feedback and monitoring  3. Social support  5. Natural consequences | 1.2. Problem solving  1.3. Goal setting (outcome)  1.4. Action planning  1.7. Review outcome goal(s)  2.2. Feedback on behavior  2.4. Self-monitoring of outcome(s) of behavior  3.3. Social support (emotional)  5.1. Information about health consequences | Problem solving   - coping plans were discussed and written with each participant in her own home after baseline data had been collected - coping plans were to identify how to cope with possible barriers towards increasing walking   Goal setting   - the average daily pedometer count over three consecutive days (at baseline before intervention) was taken and used to set a target of achieving a 10%, 15% or 20% increase in steps during the first month   Action planning   - individualized activity action plans designed to increase participants' PA levels, mainly through walking   Review outcome goal(s)   - if the participant met her target step count, it was increased again after the first and second months - if participants had not met her target, 10%, 15% or 20% was added to the average number of steps they achieved over the three day period preceding the telephone call   Feedback on behavior   - activity tracker monitored and provided information on daily step counts   Self-monitoring   - daily activity diary to complete with logs of either pedometer count or time spent walking outdoors   Social support   - each participant was contacted by telephone once a week for the first month and then fortnightly thereafter until the end of the study to provide motivation, encouragement and to troubleshoot any problems   Information about health consequences   - each participant was given advice about the health benefits of increasing PA both verbally and in pamphlet form after collection of baseline data |
| Thomas et al. (2012) | 1. Goals and planning  2. Feedback and monitoring  4. Shaping knowledge | 1.2. Problem solving  1.3. Goal setting (outcome)  1.4. Action planning  1.7. Review outcome goal(s)  2.2. Feedback on behavior  2.3. Self-monitoring of behavior  2.7. Feedback on outcome(s) of behavior  4.1. Instruction on how to perform the behavior | Problem solving   - participants received motivational meetings to provide assistance in overcoming any potential hurdles that might have arisen while implementing the behavior modification   Goal setting   - participants were asked to increase the number of steps they take during a normal day by an extra 3500 steps a day (three to five times a week) - research staff set walking goals with the participants on a weekly basis using a structured protocol (during phone calls)   Action planning   - at the baseline visit, all participants received group-based face-to-face counseling and advice on how to increase energy expenditure via integration of PA into their daily routines and basic strategies for starting (e.g., start slowly and work the exercise into the daily routine)   Feedback on behavior   - if the participants had walked, the feedback was supportive of the amount of walking the individual has accomplished - if the participant had not walked, the feedback was supportive of future attempts   Self-monitoring   - participants provided the interviewer information about the frequency, time, and distance they had walked based on their weekly walking data recorded in their PA diaries   Instruction on how to perform the behavior   - at the baseline visit, all participants received group-based face-to-face counseling and advice on how to increase energy expenditure via integration of PA into their daily routines |
| Yamada et al. (2012) | 1. Goals and planning  2. Feedback and monitoring | 1.3. Goal setting (outcome)  1.7. Review outcome goal(s)  2.2. Feedback on behavior  2.4. Self-monitoring of outcomes of behavior  2.7. Feedback on outcome(s) of behavior | Goal setting   - participants were instructed to increase their mean daily steps by 10% each month   Review outcome goal(s)   - written activity logs were monthly averaged to determine whether the participants were achieving their step goal   Feedback on behavior   - a sheet for brief feedback and setting the number of daily steps was mailed to all participants to evaluate the recorded calendar monthly   Self-monitoring   - participants were asked to record the date on the calendar and steps taken at the end of each day |
